# Redox regulation of the insulin signalling pathway

**DOI:** 10.1016/j.redox.2021.101964

**Published:** 2021-04-02

**Authors:** Claudia Lennicke, Helena M. Cochemé

**Affiliations:** a MRC London Institute of Medical Sciences, Du Cane Road, London, W12 0NN, UK; b Institute of Clinical Sciences, Imperial College London, Hammersmith Hospital Campus, Du Cane Road, London, W12 0NN, UK

**Keywords:** H_2_O_2_, Redox regulation, ROS, Insulin signalling, Cysteine post-translational modification, FOXO, NRF2, Type-2 diabetes, ARE, antioxidant response element, BCNU, bis-chloroethyl nitrosourea, EGF, endothelial growth factor, EGFR, endothelial growth factor receptor, FOXO, forkhead box class O, G6PD, glucose-6-phosphate dehydrogenase, GK, glucokinase, Grx, glutaredoxin, GSH, glutathione, GSSG, glutathione disulphide (oxidised glutathione), GPX, glutathione peroxidase, GR, glutathione reductase, GST, glutathione *S*-transferase, GSK3β, glycogen synthase kinase-3β, H_2_O_2_, hydrogen peroxide, HFD, high fat diet, IGF, insulin-like growth factor, IR, insulin receptor, IRS, insulin receptor substrate, keap1, kelch-like ECH-associated protein 1, KO, knock-out, NOX, NADPH oxidase, NRF2, nuclear factor erythroid 2-related factor 2, PDK, 3-phosphoinositide-dependent protein kinase, PI3K, phosphoinositide 3-kinase, PIP_2_, phosphatidylinositol 4,5-bisphosphate, PIP_3_, phosphatidylinositol 3,4,5-trisphosphate, PKB, protein kinase B (also known as AKT), PDGF, platelet-derived growth factor, PDGFR, platelet-derived growth factor receptor, PTEN, phosphate and tensin homolog, PTM, post-translational modification, PTP, protein tyrosine phosphatase, ROS, reactive oxygen species, SOD, superoxide dismutase, SREBP, sterol regulatory element-binding protein, Trx, thioredoxin, TrxR, thioredoxin reductase, T2D, type-2 diabetes, WT, wild-type

## Abstract

The peptide hormone insulin is a key regulator of energy metabolism, proliferation and survival. Binding of insulin to its receptor activates the PI3K/AKT signalling pathway, which mediates fundamental cellular responses. Oxidants, in particular H_2_O_2_, have been recognised as insulin-mimetics. Treatment of cells with insulin leads to increased intracellular H_2_O_2_ levels affecting the activity of downstream signalling components, thereby amplifying insulin-mediated signal transduction. Specific molecular targets of insulin-stimulated H_2_O_2_ include phosphatases and kinases, whose activity can be altered via redox modifications of critical cysteine residues. Over the past decades, several of these redox-sensitive cysteines have been identified and their impact on insulin signalling evaluated. The aim of this review is to summarise the current knowledge on the redox regulation of the insulin signalling pathway.

## 1 Introduction

Insulin, an anabolic peptide hormone secreted by pancreatic β-cells, is a key regulator of important metabolic processes such as glucose and lipid homeostasis, as well as a determinant of longevity [1,2]. The actions of insulin are mediated by the so-called insulin signalling pathway, initiated by the binding of insulin to its receptor, which triggers a sequence of intracellular phosphorylation events [2]. The insulin pathway maintains metabolic homeostasis by redirecting post-prandial glucose into muscle and adipose tissues, and by suppressing glucose production in the liver. Furthermore, insulin regulates the generation of energy storage, such as glycogen and triacylglycerides [3]. In this context, insulin resistance is defined as the decreased ability of a tissue to adequately respond to the actions of insulin, which is a risk factor for developing type-2 diabetes (T2D) [4]. The worldwide prevalence of T2D has grown concerningly over recent decades, and is estimated to increase further from 9.3% in 2019 (463 million people) to 10.9% by 2045 (700 million people) [[4], [5], [6]]. Furthermore, T2D is an important risk factor for the development of co-morbidities including cardiovascular and kidney diseases, resulting in reduced quality of life [7]. An undeniable connection exists in Western societies between behaviour, diet, obesity and T2D. The growing incidence of T2D is linked, at least partly, to decreased energy expenditure due to our increasingly sedentary lifestyles, and to increased consumption of processed foods and drinks containing high levels of sugar [8,9]. The pathological hallmarks of T2D in mammals include: **i)** the resistance of peripheral tissues to insulin signals, leading to **ii)** hyperglycemia and compensatory hyperinsulinemia, and **iii)** impaired/abnormal insulin secretion by pancreatic β-cells [4]. Most individuals with T2D are obese, and obesity itself can cause insulin resistance [4].

Several studies have implicated the involvement of oxidative stress in the development of insulin resistance and T2D. Oxidative stress arises from the aberrant production or defective scavenging of reactive oxygen species (ROS), leading to damage of macromolecules [10]. For example, ROS associated with several abnormalities, such as hyperglycemia, non-enzymatic glycosylation, inflammation and/or dyslipidemia, may cause decreased insulin expression and/or an impaired response to the insulin signal [[11], [12], [13]]. Thus, the increased supply of energy substrates and the inflammatory environment under T2D conditions are thought to result in the excessive generation of mitochondria-derived ROS that suppress the insulin signalling cascade and thereby promote the development of insulin resistance [13,14].

Besides these harmful and cell damaging high levels of ROS, lower doses of ROS, in particular hydrogen peroxide (H_2_O_2_), play essential roles in fine-tuning the insulin signalling pathway and are therefore indespensible for its optimal functioning. Several phosphatases, including those counteracting the insulin-stimulated phosphorylation cascade, contain critical cysteine residues at their active site that can be oxidised in response to H_2_O_2_, resulting in their inactivation. Similarly, several kinases that mediate insulin signalling are prone to redox regulation.

In this review, we summarise the current state of knowledge on redox signalling events that contribute to regulating the insulin signalling pathway. Furthermore, we show that key redox-active cysteine residues are evolutionarily conserved from invertebrates to humans, which is consistent with their significant physiological role.

## 2 Redox signalling

### 2.1 Cellular sources of ROS

ROS is a commonly used term that includes both short-lived and more stable products from the reduction of molecular oxygen (O_2_). The step-wise transfer of single electrons during O_2_ reduction results in the formation of the superoxide radical anion (O_2_^•–^), as well as non-radical species such as H_2_O_2_ [15]. In the presence of iron ions, H_2_O_2_ can form the hydroxyl radical (^•^OH), which is highly reactive (diffusion-limited) and therefore more implicated in oxidative damage rather than redox signalling. Superoxide is generated ‘accidently’ by the mitochondrial respiratory chain as a by-product of aerobic metabolism [16]. Moreover, superoxide can be produced deliberately by the NADPH oxidase (NOX) family of enzymes in response to stimuli, including growth factors such as insulin [17]. H_2_O_2_ is the most abundant ROS in eukaryotes with a cellular steady-state concentration of ~1–10 nM [15]. H_2_O_2_ is formed from superoxide by the action of superoxide dismutase (SOD) enzymes [15]. H_2_O_2_ can also be produced directly, for instance by xanthine oxidase in purine catabolism or by the endoplasmic reticulum oxidoreduction ERO1 during disulphide bond formation by the protein folding system [18].

While excessive formation of ROS is associated with the development of many diseases in humans including T2D, it has become increasingly apparent over recent years that low levels of specific ROS, particularly H_2_O_2_, are required for normal cellular function, and are involved in the regulation of many physiological processes such as signal transduction, cell differentiation and proliferation [15]. H_2_O_2_ is formed enzymatically by NOXs together with SODs in response to growth factor signals, e.g. insulin. This localised transient H_2_O_2_ burst is essential for optimal tyrosine-phosphorylation-dependent signalling events by modifying the activity of kinases. These include the insulin receptor (IR) kinase domain itself (see Section 4.2), as well as phosphoinositide 3-kinase (PI3K) and AKT. Furthermore, the activity of phosphatases, such as PTEN, PTP1B and PP2A, that counteract insulin signalling by dephosphorylating and thereby inhibiting insulin-responsive kinases, is modulated by H_2_O_2_ (see Section 4.3). Thus, the localisation and levels of ROS have an impact on the progression of the insulin signalling cascade.

### 2.2 Cysteine-based redox modifications

**Fig. 1:**
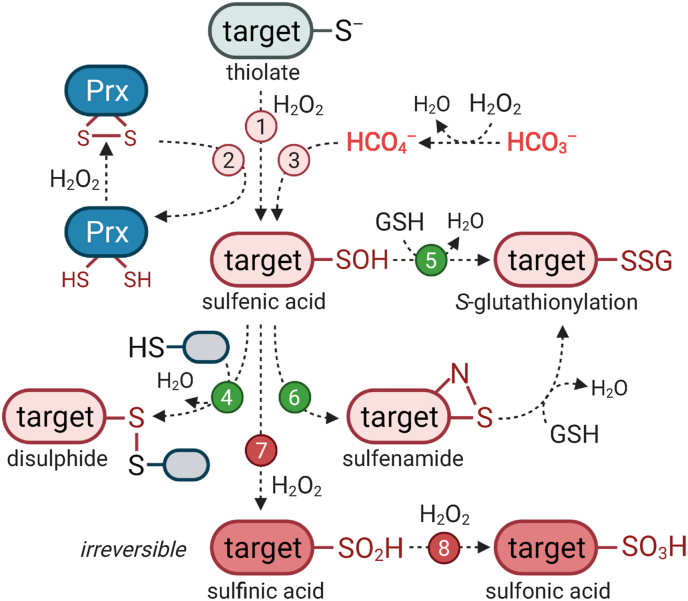
Potential redox modifications of cysteines by H_2_O_2_. Cysteine thiols (R–SH) are partially deprotonated at physiological pH to the thiolate state (R–S^–^). In the presence of H_2_O_2_, the thiolate can undergo initial oxidation to form sulfenic acid (R–SOH), through a range of mechanisms: (**1**) direct oxidation, (**2**) oxidation mediated by a highly redox-reactive second protein such as peroxiredoxin (Prx), or (**3**) after exposure to highly reactive compounds such as peroximonocarbonate (HCO_4_^−^), which is spontaneously generated in a reversible reaction between H_2_O_2_ and bicarbonate (HCO_3_^−^) (see also Fig. 2). Sulfenic acid is relatively reactive and can form inter- or intra-molecular disulphides with a second adjacent thiol (R–SS–R) (**4**). Alternatively, the sulfenic acid can react with a low molecular weight thiol, e.g. GSH results in *S*-glutathionylation (R-SSG) (**5**). In some proteins (e.g. PTP1B), an intermediate redox formation occurs, where the sulfenic acid reacts rapidly with a serine in close proximity to form a sulfenamide (R–SN) that can be further *S*-glutathionylated (**6**). These redox modifications are reversible and can be reduced back to the initial thiolate state by cellular antioxidant systems. However, under conditions of oxidative stress, the sulfenic acid can form higher oxidation states: (**7**) sulfinic acid (R–SO_2_H) and (**8**) sulfonic acid (R–SO_3_H), which are both irreversible modifications.

Amongst the various ROS, H_2_O_2_ exhibits low overall reactivity, but relatively high selectivity towards specific thiol groups (R–SH) of cysteine residues (Fig. 1). Therefore, redox-based post-translational modification (PTM) of cysteines in target proteins can modulate a wide range of biological processes [15,19]. The cysteine proteome serves as an adaptive interface between the genome and the external environment of an organism. The reversible oxidation of cysteines involved in redox signalling processes depends on their location at the protein surface, and hence their accessibility to oxidants and antioxidants. The human genome encodes ~214,000 cysteine residues - a frequency lower than expected (~2.26% versus 3.28%) [20]. Cysteines play important functions as structural disulphides in mediating protein folding, or as catalytic active sites in enzymes such as phosphatases and proteases. This biochemical reactivity explains why cysteine residues are highly evolutionary conserved, but selected against if non-functional [21,22].

Cysteines in their deprotonated thiolate form (R–S^–^) can undergo oxidative modification by H_2_O_2_ to form a sulfenic acid (R–SOH), intra-/inter-molecular disulphides (R–SS–R), or higher oxidation states, such as sulfinic (R–SO_2_H) and sulfonic acids (R–SO_3_H). Cysteine oxidation to sulfenic acid and disulphides is completely reversible. The reduction of sulfenic acids is mediated either by the glutaredoxin (Grx) and thioredoxin (Trx) systems that allow rapid enzymatic reduction, or by free low molecular weight thiols such as glutathione (GSH) (Fig. 1) [23]. GSH is a tripeptide synthesised from the amino acids glutamate, glycine and cysteine within the cytosol, and transported to other cellular compartments. The synthesis of GSH is enzyme-catalysed in two ATP-dependent reactions that are strongly inducible by the transcription factor NRF2 [24] (see Section 5.1).

As a low molecular weight thiol-containing antioxidant, GSH is present in all eukaryotic cells [25]. GSH directly scavenges free radicals by donating two electrons when two GSH molecules react together to give an oxidised disulphide form (GSSG). Furthermore, GSH is necessary for the function of glutathione peroxidase (GPX) enzymes, which detoxify H_2_O_2_, lipid peroxides, or other organic peroxides [26]. *S*-Glutathionylation can occur in two ways: **i)** as a nucleophilic attack by GSH on an oxidised thiol, or **ii)** by the reverse reaction. This process can happen either enzymatically catalysed or uncatalysed, and occurs under conditions of both oxidative and nitrosative stress [15]. *S*-Glutathionylation acts as a reversible mechanism to modulate the function of target proteins (in most cases, associated with their inactivation), as well as to protect cysteines from irreversible hyper-oxidation that would lead to permanent damage [15,24,27].

The redox-reactivity of each cysteine varies considerably depending on its structural context and local environment [28]. Analysing the redox proteome (i.e. redoxome) of several mouse tissues revealed that redox-sensitive cysteine residues exist in a local environment that tunes the side-chain for oxidative modifications [29]. A common amino acid signature was enriched surrounding highly modified cysteine residues: selection against acidic amino acids and selection for basic amino acids. This specific spanning signature can be explained by the effects of the proximal charge on the cysteine side-chain. The thiol/thiolate equilibrium is sensitive to electrostatic changes, with a positively charged residue (e.g. arginine) stabilising the negatively charged thiolate [29]. Conversely, the negative charge of a phosphorylated side-chain is predicted to antagonise cysteine oxidation, and indeed the % cysteine modification was negatively correlated with the propensity of phosphorylation proximal to this site [29]. In contrast, no correlation was observed in adipocytes treated with the drugs BCNU (bis-chloroethyl nitrosourea) and auranofin, suggesting that changes in protein phosphorylation in response to simultaneous thioredoxin reductase (TrxR) and glutathione reductase (GR) inhibition were unlikely a direct result of oxidation of these proteins. Thus, a global protein phosphorylation change in response to oxidative events might be the result of redox-dependent changes in the activity of upstream kinases [30]. In this sense, the oxidation of a cysteine residue that is necessary for catalytic function, e.g. in cysteine-dependent phosphatases, would interfere with the formation of the thiol-phosphate intermediate during the dephosphorylation process, therefore maintaining the target protein in a phosphorylated state. A similar scenario and direct redox regulation of cysteine-dependent kinases is possible, where oxidation for signalling purposes might involve structural re-configuration, resulting in enhanced, altered or decreased kinase activity [31].

**Fig. 2:**
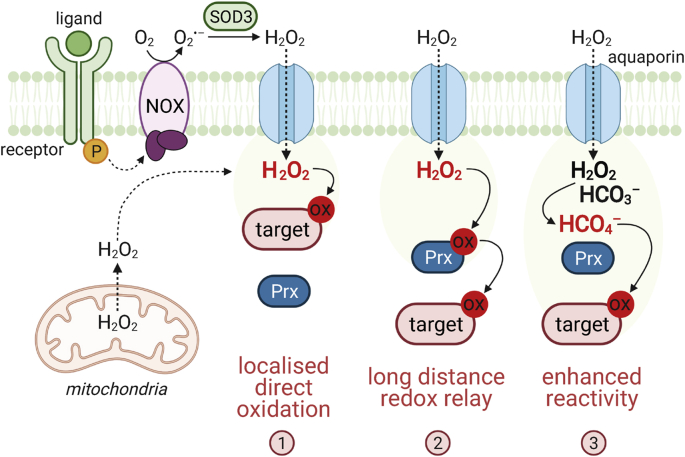
Mechanisms of redox signalling. Activation of growth factors leads to the induction of superoxide anion (O_2_^•–^) formation by NOX enzymes, which is subsequently converted to H_2_O_2_ catalysed by extracellular SOD3. H_2_O_2_ enters cells by facilitated passive diffusion via aquaporins, which results in a localised intracellular H_2_O_2_ burst. Alternatively, superoxide is generated in mitochondria as a by-product of respiratory metabolism and converted to H_2_O_2_ by SOD2 in the matrix. The reactivity of H_2_O_2_ with antioxidant enzymes such as peroxiredoxins (Prx) is several orders of magnitude higher than towards the thiol of a ‘normal’ target protein. Therefore, several models have been proposed to explain how redox modifications of target proteins are possible. The target protein is located in close proximity to the site of H_2_O_2_ formation, enabling close-range direct oxidation (**1**). In the redox relay model, a highly reactive sensor, e.g. Prx, acts as a redox messenger protein, transmitting the oxidation to a subsequent target cysteine, which allows longer-range signalling (**2**). The reactivity of H_2_O_2_ can be enhanced by interaction with cellular bicarbonate (HCO_3_^−^) to form the highly reactive peroxymonocarbonate anion (HCO_4_^−^), that can directly oxidise the target (**3**).

Superoxide anions and H_2_O_2_ are efficiently removed by cellular antioxidant systems (e.g. SOD, peroxiredoxin (Prx), GPX, catalase), thus contributing to generally low intracellular H_2_O_2_ levels. Given the high cellular abundance of antioxidant enzymes such as Prx and their strong reactivity with H_2_O_2_ [32], it is currently debated how the oxidation of a target cysteine can occur by H_2_O_2_ in this environment to allow redox signalling. So far, three main models have been established: **i)** localised formation of a H_2_O_2_ burst that allows the direct oxidation of a less reactive target protein [33], **ii)** indirect oxidation via a redox relay mediated by a highly redox-sensitive protein, such as Prx [34], and **iii)** formation of more reactive molecules such as peroxymonocarbonate (HCO_4_^−^) [35,36] (Fig. 2).

Similar to other PTMs (e.g. phosphorylation, acetylation or ubiquitination), reversible redox modifications can alter the activity, function and/or localisation of the respective target protein. Therefore, redox signalling is a highly dynamic process, depends on the cellular balance between oxidants and antioxidants, and is coupled to stress levels and energy status.

## 3 Signalling by insulin

Insulin plays an essential role in controlling nutrient and metabolic homeostasis. Insulin promotes glucose storage in the liver and glucose uptake into fat and muscle cells via the glucose transporter GLUT4. In response to insulin, GLUT4 is translocated from the cytoplasm to the plasma membrane. Mice lacking GLUT4 are insulin resistant [37]. The precise mechanism whereby insulin leads to the translocation of GLUT4 is still not fully understood, but the PI3K/AKT pathway is known to play an essential role.

The insulin receptor (IR) is a α2β2 heterotetrameric glycoprotein belonging to the receptor tyrosine kinase superfamily, with 20 sub-families described in humans, based on sequence homology, structure and ligand affinity [38,39]. Besides the IR, other members of the receptor tyrosine kinase superfamily include endothelial growth factor receptor (EGFR) with its ligand EGF, and platelet-derived growth factor receptor (PDGFR) with its ligand PDGF [39].

Binding of insulin to the IR extracellular domain induces a conformational change of the IR that enables binding of ATP at its cytoplasmic β domain, resulting in rapid IR auto-phosphorylation. This intracellular activation of specific tyrosine residues in the IR kinase domain is essential for mediating all the metabolic effects of insulin, starting with the recruitment of downstream signalling molecules such as insulin receptor substrates (IRSs) [2]. Tyrosine phosphorylation of IRS enables the binding of various proteins containing an SH2 domain, importantly PI3K, which phosphorylates phosphatidylinositol 4,5-bisphosphate (PIP_2_) to phosphatidylinositol 3,4,5-trisphosphate (PIP_3_) at the plasma membrane. Increased intracellular levels of PIP_3_ attract 3-phosphoinositide-dependent protein kinase 1 (PDK1), a serine/threonine kinase, and protein kinase B (PKB) also known as AKT. Phosphorylated PDK1 in turn phosphorylates and thereby activates AKT, which is the key player in insulin-mediated regulation of metabolism and gene expression [40]. The AKT family consists of three isoforms (AKT1,2,3) in mammals, but AKT2 specifically regulates insulin-dependent glucose homeostasis [41]. Transgenic AKT2-knock-out (KO) mice develop insulin resistance and a T2D-like phenotype [42]. Moreover, significant defects in glucose uptake were observed in cultured adipocytes obtained from mice lacking AKT2 [43]. The missense loss-of-function mutation AKT2-Arg274His leads to hyperinsulinemia and insulin resistance in humans [44], highlighting the importance of AKT2 in mediating insulin signalling. Conversely, the activating mutation AKT2-Glu17Lys within the PH domain of AKT2, required for docking to PIP_3_ at the plasma membrane, results in hypoinsulinemia [45]. Over-expression of this mutant in 3T3-L1 adipocytes produced non-insulin-dependent membrane localisation of GLUT4, AKT2 activation even under serum-starved conditions in HeLa cells, and a tonic nuclear exclusion of the AKT target FOXO1 from the nucleus [44,45], showing the importance of the PH domain and PIP_3_ in activating AKT2.

AKT activation induces glycogen storage via inhibition of glycogen synthase kinase 3β (GSK3β), and promotes the uptake of glucose via translocation of GLUT4 vesicles to the cell membrane [2]. Although the PI3K/AKT pathway regulates long-term effects such as cellular differentiation and proliferation, its primary goal is the acute regulation of glucose metabolism in insulin-target tissues.

Several mechanisms modulate insulin signalling, including the regulation of ligand production and availability, the endosomal internalisation and downregulation of the IR, the degradation of IRSs, and the dephosphorylation of insulin targets by specific phosphatases. In this context, the protein tyrosine phosphatase 1B (PTP1B) is described to dephosphorylate the IR kinase domain, PI3K and AKT, thus acting as an important negative regulator of insulin signalling [46]. PTP1B-deficient mice are characterised by increased insulin sensitivity and are resistant to high fat diet (HFD)-induced obesity [47,48]. Futhermore, PTP1B polymorphisms in humans are associated with morbid obesity [49].

Therefore, optimal regulation of glucose metabolism by insulin requires tight regulation, and a balance of phosphorylation and dephosphorylation events. In the following sections, the redox-mediated fine-tuning of key components from the insulin cascade will be described.

## 4 Insulin-mediated H_2_O_2_ generation regulates insulin signalling

Chronically high ROS associated with hyperglycemia are recognised to have pathophysiological roles in the progression of T2D, e.g. impairment of β-cell function, or the development of further co-morbidities such as vascular complications. Studies in healthy humans and rodents further revealed that the adipose tissue experiences oxidative stress as a result of excessive caloric intake leading to the development of insulin resistance [50,51]. Using a model of physiologically derived oxidative stress by inhibiting TrxR and GR simultaneously in adipocytes, >2000 genes were found to be differentially expressed compared to untreated cells [10]. Interestingly, this response shared many similarities with changes observed in insulin resistance models. Providing these cells with an antioxidant induced only minor transcriptional changes, but rescued the insulin resistance. This indicates that the transcriptional changes observed in response to oxidative stress are not the cause of insulin resistance. Thus, oxidative stress must have effects in addition to transcriptional changes to cause insulin resistance [10].

In this context, it may seem ‘paradoxical’ that the over-expression of antioxidant enzymes leads to the development of insulin resistance in mice [52], and that growth factors such as insulin stimulate the generation of localised ROS, which play a role in facilitating downstream insulin signalling [[53], [54], [55]]. The insulin-mediated production of H_2_O_2_ was first described in 1979 in rat adipocytes and is coupled with increased glucose metabolism [56]. Blocking the insulin-stimulated cellular production of H_2_O_2_ completely inhibits PI3K activity and dramatically reduces insulin-stimulated AKT activation by ~50% [57].

### 4.1 Insulin-mediated H_2_O_2_ formation via NOX enzymes

NOXs are pro-oxidant enzymes with the main cellular function of producing ROS. Cell membrane-associated NOX enzymes transfer electrons from intracellular NAD(P)H across cell membranes to O_2_ thereby producing superoxide, which is dismutated to the ‘second messenger’ H_2_O_2_ by the action of SODs. H_2_O_2_ enters the cell facilitated by aquaporins to exert its signalling function [24] (Fig. 2).

NOX4, located at the plasma membrane, has been shown to convey several signalling events in different insulin-dependent tissues, e.g. vascular smooth muscle, endothelial cells, fibroblasts and hepatocytes [58]. NOX4 was also identified as the NOX isoform in adipocytes responsible for H_2_O_2_ production in response to insulin stimulation [59]. The expression of NOX4 constructs that lack the NADPH or FAD/NADPH cofactor-binding domains in differentiated adipocytes attenuated H_2_O_2_ production in response to insulin, tyrosine phosphorylation of the IR kinase domain and IRS proteins, and diminished activation of the PI3K/AKT pathway and downstream insulin events, e.g. glucose uptake [59].

Over-expression of PTP1B, a negative regulator of insulin signalling, inhibited insulin-stimulated tyrosine phosphorylation of the IR. This effect could be efficiently reversed by simultaneous over-expression of NOX4 [59]. In contrast, NOX2-KO in cardiomyocytes blocked the insulin-mediated phosphorylation of AKT and mTOR, while these responses were unaffected in cardiomyocytes obtained from NOX4-KO mice. However, the effects of insulin on contractility were lost in cardiomyocytes from NOX4-KO mice, but retained in the NOX2-KO mutant [60].

NOX4 is a regulator of metabolic homeostasis as the absence of NOX4-mediated ROS production results in adipose tissue hypertrophy, and sensitivity towards diet-induced hepatosteatosis and obesity [61]. On the other hand, elevated NOX4 levels have been observed in the adipose tissue in rodent models of obesity, as well as in humans with extreme insulin resistance [62,63]. Pharmacological inhibition of NOX4 in HFD-induced glucose-intolerant C57BL/6 mice counteracted non-fasting hyperglycemia and impaired glucose tolerance without any change in peripheral insulin sensitivity [64]. Thus, the manipulation of redox status and its effects on the modulation of signalling pathway components might promote insulin signalling but may also contribute to obesity. Further, these data support recent observations that adequate H_2_O_2_ production is vital for the maintenance of whole-body homeostasis [61].

### 4.2 IR kinase activity is dependent on H_2_O_2_

**Fig. 3:**
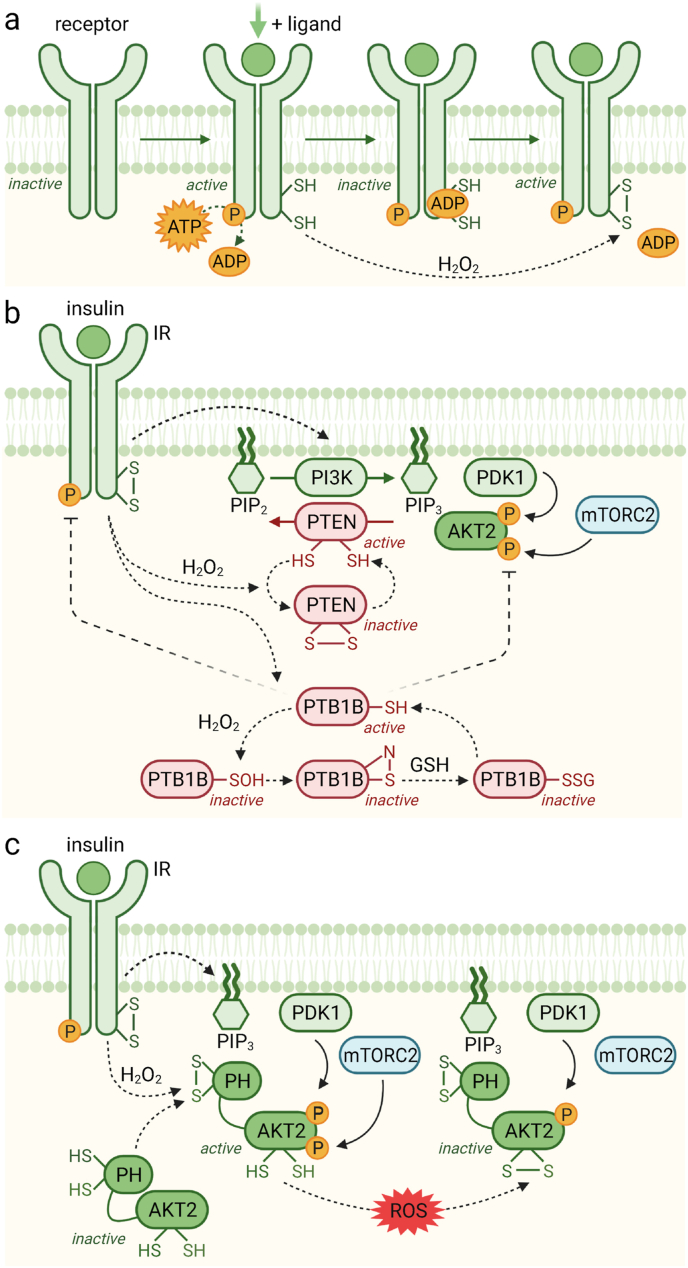
Models for H_2_O_2_-dependent regulation of the insulin signalling cascade. (**a**) The binding of insulin to the insulin receptor (IR) leads to IR auto-phosphorylation, which generates ADP. This ADP can act as a negative feedback inhibitor of IR kinase activity. Binding of insulin to the IR also results in the activation of NOX enzymes and the formation of localised H_2_O_2_, which oxidises two redox-sensitive cysteine residues (Cys1245 and Cys1308) in the IR kinase domain, that interferes with the inhibitory effect of ADP, thereby prolonging IR signalling activity. (**b**) Active IR stimulates PI3K, a kinase that phosphorylates PIP_2_ to PIP_3_. Enhanced PIP_3_ levels in the plasma membrane attract AKT and PDK1, bringing both enzymes in contact, and allowing PDK1 to phosphorylate AKT2 at Thr308. In parallel, mTORC2 phosphorylates AKT2 at Ser473, leading to full activation. This process is counteracted by phosphatases: PTEN dephosphorylates PIP_3_, whereas PTP1B dephosphorylates the IR and AKT2, thus terminating insulin signalling. Both phosphatases are susceptible to redox regulation and are inactive when oxidised. IR-mediated H_2_O_2_ leads to the formation of a disulphide between Cys77 and Cys124 in PTEN, whereas PTP1B undergoes sulfenamide formation and *S*-glutathionylation at Cys215. (**c**) AKT2 activity is also fine-tuned by redox mechanisms. Insulin-stimulated H_2_O_2_ generation leads to the formation of a disulphide located in the PH domain of AKT2 (Cys60-Cys77), which enhances its recruitment to the plasma membrane, and subsequent activation by phosphorylation. However, high levels of ROS lead to the formation of a second inhibitory disulphide (Cys297-Cys311) located in the kinase domain of AKT2, resulting in diminished activity.

Direct modulation of IR activity by H_2_O_2_ was observed in early studies with thiol-reactive agents, e.g. iodoacetamide, *N*-ethylmaleimide, and maleimidobutyrylbiocytin [65]. Furthermore, the treatment of cells with the GR inhibitor BCNU resulted in decreased IR β-subunit free thiol groups [66]. The first evidence showing that specific cysteine residues might play a role in IR activity came from a study using CHO and NIH3T3 cells transfected with human IR-Cys1138Ala mutants which were found to be functionally defective [67]. Later, using purified recombinant fragments of the IR kinase domain, it was shown that H_2_O_2_ modulates IR kinase activity and promotes its auto-phosphorylation in the presence of otherwise inhibitory physiologically relevant concentrations of ADP [68]. This enhancement of IR auto-phosphorylation by H_2_O_2_ was unchanged in the Cys1234Ala mutant, decreased in the Cys1245Ala mutant, and completely inhibited in the Cys1308Ala mutant [68] (Fig. 3a, Table 1). In skeletal muscle tissue, the generated ADP is rapidly converted to ATP by the enzyme creatine kinase via utilisation of phosphocreatine. In this sense, enhanced IR kinase activity under high ADP levels and in response to H_2_O_2_ is expected to play a role in tissues with lower abundance of creatine kinase, e.g. adipose tissue.

**Table 1:** Examples of redox-regulated components involved in mediating insulin signalling.

Protein	Category	Residue (location)	Modification	Oxidant	Interaction	Effect	Ref.
Insulin receptor	Kinase	**Cys1245** (kinase domain) **Cys1308** (kinase domain)	Intramol. disulphide *S*-glutathionylation	H_2_O_2_	GSH	Activation, promotes the release of inhibitory ADP from the kinase domain	[68]
PTEN	Phosphatase	**Cys77** **Cys124** (catalytic active centre)	Intramol. disulphide	H_2_O_2_		Inactivation	[69,70,71]
PTP1B	Phosphatase	**Cys215** (catalytic active centre)	Sulfenamide/*S*-glutathionylation	H_2_O_2_	GSH	Inactivation	[72]
AKT2	Kinase	**Cys60** (PH domain) **Cys77** (PH domain)	Intramol. disulphide	H_2_O_2_		Activation, promotes AKT2 recruitment to the PM	[30]
		**Cys124** (linker domain)	Intramol. disulphide	H_2_O_2_/catalase inhibition		Inactivation	[73]
		**Cys297** (activation loop) **Cys311** (activation loop)	Intramol. disulphide	H_2_O_2_ BCNU/auranofin		Inactivation	[74,75] [30]
FOXO1	TF	**Cys612**	Intermol. disulphide	Basal conditions	P300/CBP, PGC1α	Full transactivation activity	[76]
				H_2_O_2_	P300/CBP	FOXO1 acetylation, cell cycle arrest and induction of pro-apoptotic genes	[77]
FOXO3	TF	**C31** **C150** (Forkhead domain)	Intermol. disulphide	H_2_O_2_	Prdx1	Decreased FOXO3 transcriptional activity under low H_2_O_2_ doses that abrogated under high doses	[78]
FOXO4	TF	**Cys477**	Intermol. disulphide	H_2_O_2_	P300/CBP	FOXO4 acetylation, cell cycle arrest and induction of pro-apoptotic genes	[79]
			Intermol. disulphide	H_2_O_2_	TNPO1	Nuclear translocation	[80]

*intramol., intramolecular; intermol. intermolecular; PM, plasma membrane; TF, transcription factor.

Together these results show that the inhibition of IR kinase activity by its product ADP is subject to redox regulation [68]. ADP binds within the catalytic active site of the IR kinase domain thereby inhibiting its activity. However, exposure to H_2_O_2_ induces the oxidation of both Cys1245 and Cys1308 residues, which results in ADP release and thereby recovery of IR activity. The precise mechanism still needs to be clarified but potentially involves the formation of a disulphide between Cys1245 and Cys1308 [68]. Interestingly, H_2_O_2_ also increases IR auto-phosphorylation in the absence of insulin. This suggests that basal IR kinase activity is dependent on the redox status in the plasma and might change under different conditions, e.g. age [68]. This hypothesis is also supported by the notion that the GR inhibitor BCNU, which alters the cellular GSH status, enhances IR activity [68].

### 4.3 Phosphatases are inhibited by insulin-induced H_2_O_2_

Purified IRs maintain their phosphorylation even after removal of insulin. Therefore, cellular phosphatases are recognised as playing a significant role in terminating and regulating IR activity and its downstream components [81,82]. Protein tyrosine phosphatases (PTPs) function as negative regulators of insulin signalling by dephosphorylating and thereby inactivating insulin targets, including the IR and AKT [46].

PTPs share a common conserved motif at their catalytic site: (I/V)H**C**XAGXXR (S/T/G), including a critical cysteine residue which is necessary for their enzymatic function [46]. This cysteine performs a nucleophilic attack on the downstream phospho-tyrosine target, exhibits a low pK_a_, and is therefore also prone to oxidation. In general, the catalytic cysteine of PTPs forms a cyclic sulfenamide with the nitrogen of a neighbouring serine residue or becomes glutathionylated upon exposure to oxidants [83,72] (Fig. 3b). An exception is PTEN where the catalytically essential cysteine forms an inhibitory disulphide with a close-by cysteine [69]. Re-activation of oxidised PTP enzymes is achieved by reduction of the catalytic cysteine by Trx [84].

*Phosphatase and tensin homolog (PTEN)* initially became famous as a tumour suppressor [[85], [86], [87]]. PTEN reverses the action of PI3K by specifically removing the phosphate attached to the 3′-hydroxyl group of PIP_3_ and thereby negatively modulates the PI3K/AKT axis [85,88]. PTEN contains a critical low pK_a_ cysteine residue. This essential Cys124 forms a disulphide with the structurally adjacent Cys71 in response to H_2_O_2_, leading to PTEN inactivation and subsequent activation of downstream signalling [69,89] (Fig. 3b, Table 1). Stimulation of macrophages with lipopolysaccharides (LPS) results in increased oxidation of PTEN. Furthermore, the stimulation of neuroblastoma cells or HEK293 cells with insulin, HeLa cells stimulated with EGF, or fibroblasts stimulated with PDGF resulted in PTEN oxidation [70,71]. In addition to disulphide formation upon oxidation, PTEN also undergoes *S*-glutathionylation in response to GSSG [90,91]. The Cys124-Cys77 disulphide bond is reversed by Trx [69], with GSH also important for reducing oxidised PTEN [90]. Besides, an increase in insulin-induced ROS generation due to PrxII deficiency results in PTEN inhibition and enhanced insulin sensitivity [92]. Further research is needed to determine the effects of redox-mediated PTEN inhibition not only from the perspective of T2D, but also in the context of cancer cells that are characterised by high levels of ROS and decreased PTEN activity.

*Protein-tyrosine phosphatase 1B (PTP1B)* is a negative regulator of insulin signalling through dephosphorylation of the IR and AKT. Permanently high activity of the H_2_O_2_-detoxifying enzyme glutathione peroxidase 1 (GPX1) has been positively correlated with the early development of insulin resistance and T2D in animal models [52,93]. Conversely, GPX1-KO mice exhibit enhanced ROS levels, increased PTEN and PTP1B oxidation, PI3K/AKT activation, and enhanced insulin sensitivity [55,94]. PTP1B–KO mice display lower circulating levels of fasting insulin, accompanied by increased IR phosphorylation in liver and muscle tissues. Moreover, these mice are more sensitive to stimulated endogenous or injected insulin as measured by glucose and insulin tolerance tests [[95], [96], [97]].

Members of the PTP superfamily act in a substrate-specific manner and employ a common biochemical mechanism for phosphate hydrolysis involving transient cysteinyl-phosphate intermediates. *In vivo*, PTPs undergo essentially two regulatory processes - they are inhibited through reversible oxidation of the active-site cysteine residue, and are activated through tyrosine phosphorylation of specific tyrosine residues located near the catalytic site [98]. Whereas the catalytic centre of PTP1B is located in the cytosol, the whole enzyme protein is anchored through a hydrophobic residue to the ER membrane [99]. An interaction exists between the IR precursor and PTP1B, indicating that PTP1B plays a crucial role in the impairment of insulin-independent phosphorylation and therefore activation of the immature IR precursor during its biosynthesis [100,101]. Furthermore, PTP1B expression is increased in pancreatic β-cells in response to ER stress, induced by nutritional overflow [102].

The catalytic domain of PTP1B contains an essential nucleophilic cysteine residue (Cys215) necessary for phosphatase activity. Due to its low pK_a_, Cys215 is much more susceptible to oxidation than other cysteine thiols [103,104]. In the presence of H_2_O_2_, Cys215 oxidation leads to the formation of an unstable sulfenic acid derivative, which reacts rapidly with close-by amino acid residues, either forming a sulfenamide by reaction with the adjacent Ser216 [72], or becoming *S*-glutathionylated catalysed by the action of glutathione-*S*-transferase (GST) [105,106] (Fig. 3b, Table 1). These reactions switch the enzyme into an inactive state [107]. Consequently, the insulin phosphorylation cascade works unimpeded when PTP1B is oxidised [98]. Interestingly, it has been shown that PTP1B is inhibited by H_2_O_2_ but not by superoxide [108], indicating that this mechanism is specific to H_2_O_2_.

Cys215 oxidation by H_2_O_2_ is unlikely to occur in cells in which PTP1B oxidation competes with the activity of detoxification enzymes such as GPX, TrxR and Prx, especially since the latter has a particularly high affinity for H_2_O_2_ [109]. However, the *in vivo* inactivation of PTP1B during insulin signalling occurs rapidly within minutes compared to *in vitro* experiments where purified PTP1B inactivation occurs at modest rate constants, indicating that the loss of enzyme activity would likely take hours [110]. More recently, bicarbonate (HCO_3_^−^) was shown to be essential for PTP1B oxidation. HCO_3_^−^ reacts with H_2_O_2_ forming peroxymonocarbonate anions (HCO_4_^−^), whose reaction with low molecular thiols is ~100 times faster compared to H_2_O_2_ [35] (Fig. 2). Therefore, cellular HCO_3_^−^ levels may dictate the total phosphotyrosine levels observed in cells after stimulation with EGF, and correlate with PTP1B oxidation [36]. Furthermore, 14-3-3 proteins are necessary to stabilise the oxidised form of PTP1B and prevent its re-activation [111].

High PTP1B activity triggers liponeogenesis by increasing the activity of protein phosphatase 2A (PPA2), and the subsequent activation of sterol regulatory element-binding protein-1c (SREBP-1c) [112]. SREBP-1c is a transcription factor that up-regulates the gene expression of fatty acid synthase (FAS) and other lipogenic enzymes [113]. Hepatic and pancreatic glucokinase (GK) play a key role in glucose metabolism and in mediating insulin secretion by β-cells. In the liver, GK transcription is activated by insulin. However, SREPB-1c is necessary for insulin-dependent GK gene expression. In this context, over-expression of the dominant positive form of SREBP-1c was shown to mimic the effects of insulin on GK [113]. These data indicate that SREBP-1c has a beneficial role in glucose homeostasis.

In contrast, SREBP-1c promotes fatty acid synthesis and lipid deposition. Since lipid storage is a major determinant of developing insulin resistance, SREBP-1c also negatively impacts glucose homeostasis [113,114]. Several studies have demonstrated that the over-expression of SREBP-1c causes a marked decrease in glucose-stimulated insulin secretion from β-cells, and this effect is accompanied by triglyceride accumulation and lipotoxicity [115,116]. Thus, redox-regulation of PTP1B may also have two different functions in the development of insulin resistance: **i)** by dephosphorylating the β-subunit of the IR and AKT, therefore resulting in direct inhibition of the insulin signalling cascade, and **ii)** by indirectly activating the lipogenic transcription factor SREBP-1c, followed by steatosis and lipotoxicity.

Overall, PTP1B is an attractive target for the treatment of insulin resistance, T2D and obesity. Ubiquitous deletion of PTP1B results in increased insulin sensitivity, improved glucose tolerance, and protection against diet-induced obesity, hence the development of redox-based strategies to stabilise PTP1B in an oxidised (and therefore inactive) state [117].

*Serine protein phosphatase 2A (PP2A)* is another member of the PTP family that negatively regulates insulin signalling by dephosphorylating AKT at Ser473. Inhibition of PP2A by small-molecule compounds leads to a dose-dependent increase in AKT phosphorylation and its downstream targets FOXO1 and GSK3α [118]. PP2A contains a critical cysteine residue within its catalytic active site, that is potentially susceptible to oxidation. To-date, studies on the redox regulation of PP2A are limited, although some reports provide evidence for reversible inhibition upon oxidation [119,120].

### 4.4 AKT2 is activated or inhibited by oxidative modifications depending on the site of action

AKT is a serine/threonine kinase acting downstream of growth factors, and is the key kinase regulating insulin signalling. Active AKT phosphorylates and thereby activates or inactivates its targets with the ultimate goal to enhance anabolic processes. AKT activation is dependent on lipids that are produced by PI3K, and its phosphorylation at two critical sites (Thr309 and Ser474) is mediated by PDK1 and mTORC2, respectively [121] (Fig. 3c).

The redox state of multiple cysteine residues in AKT is important for regulating its activity. AKT2, the AKT isoform that is highly enriched in insulin-target tissues [122], contains two cysteine residues (Cys297 and Cys311) within its activation loop, that form an intramolecular disulphide upon oxidation to inhibit AKT2 activity. Interestingly, these cysteine residues are conserved in the other two AKT isoforms, AKT1 and AKT3, but only modulate the activity of AKT2 [74]. The inhibitory disulphide can be resolved by the Grx system [75]. In addition, Cys124 and Cys311 in AKT2 can undergo *S*-glutathionylation. The redox regulation of Cys124 has been described in PDGF-stimulated NIH3T3 cells as an AKT2 inhibition site [73] (Fig. 3c, Table 1).

Cys77 of AKT2 is oxidised in response to BCNU/auranofin treatment, associated with hyper-phosphorylation at Thr309 [30]. Several substrates of AKT were also found to be phosphorylated, indicating that AKT2 Cys77 is active when oxidised. However, other AKT targets exhibited decreased phosphorylation, while some didn't change in response to oxidative stress, showing that not all AKT substrates are influenced by oxidative stress in the same manner. Immuno-precipitation and proteomic approaches revealed that Cys60 and Cys77 of AKT (both located in the PH domain) form a disulphide that acts as a redox switch regulating AKT binding to PIP3 and activation [30]. The PH domain is essential for AKT recruitment to the plasma membrane and subsequent phosphorylation at Thr309 (required for ordering the activation loop and substrate binding) and at Ser474 (to control the positioning of key catalytic residues) in AKT2 [123] (Fig. 3c, Table 1). Insulin stimulates PI3K resulting in the accumulation of PIP_3_ at the plasma membrane and enhanced binding of inactive cytosolic AKT via its PH domain [66]. Inside the cell, active AKT is predominantly membrane-bound and upon dissociation from the plasma membrane, the PH domain promotes AKT dephosphorylation [124]. Thus, AKT is controlled as a PIP_3_-sensitive switch, which couples membrane binding to kinase activation [124]. In line with this model, a recent study reported that insulin increases the membrane translocation of AKT2 but not AKT1 in skeletal muscle cells [121].

Phosphorylation of AKT2 at Thr309 was found to be enhanced in response to oxidative stress, a phenomenon that was impaired in an AKT2-Cys60/77Ser mutant stimulated with insulin, implying that either Cys60, Cys77 or both are necessary for insulin-stimulated AKT activation even in the absence of oxidative stress [30], since the Cys60-Cys77 disulphide is formed not only under conditions of oxidative stress but also upon insulin stimulation. This suggests that these residues are sensitive to physiological H_2_O_2_ levels [30]. Enhanced formation of the Cys60-Cys77 disulphide in response to insulin increases the affinity of AKT to PIP_3_, thus promoting its translocation to the membrane and phosphorylation [30]. As the PH domain interacts with and inhibits the kinase domain [124], the redox state of the redox-sensitive cysteines Cys60 and Cys77 within the PH domain may regulate this interaction [30].

Cys124 in the linker region of AKT2 has been identified as another regulatory cysteine, which is isoform-specific to AKT2 and not conserved in AKT1/3. Cys124 forms a sulfenyl group in response to H_2_O_2_ treatment, leading to the inhibition of kinase activity. Thus, H_2_O_2_ can enhance or decrease the activity of AKT2, depending on its levels. Together, the combinations of oxidative modifications to Cys60, Cys77, Cys124, Cys297 and Cys311 may serve to control the amplitude of AKT activity during signalling [73]. Overall, the insulin-induced formation of H_2_O_2_ results in the activation of AKT, the master regulator of insulin signalling.

## 5 The antioxidant effects of insulin

As described above, insulin exerts pro-oxidant functions via activation of NOX enzymes and subsequent generation of H_2_O_2_, which is required for optimal activation and functioning of the insulin signalling (PI3K/AKT) pathway. In addition to the important role of insulin in maintaining glucose homeostasis, insulin also possesses antioxidant effects to protect cells against oxidative stress and oxidative stress-related diseases. Many studies have investigated the antioxidant and cytoprotective functions of insulin. Insulin treatment recovers the cellular GSH/GSSG pool by upregulating the expression and activity of several enzymes with an antioxidant function, including glutamate-cysteine ligase (GCL), GR, glucose-6-phosphate dehydrogenase (G6PD), GSTs, TrxR, SOD and catalase (reviewed in Ref. [125]). Notably, these enzymes are downstream targets of the transcription factor nuclear factor erythroid 2-related factor 2 (NRF2).

### 5.1 Insulin signalling enhances the activity of NRF2

**Fig. 4:**
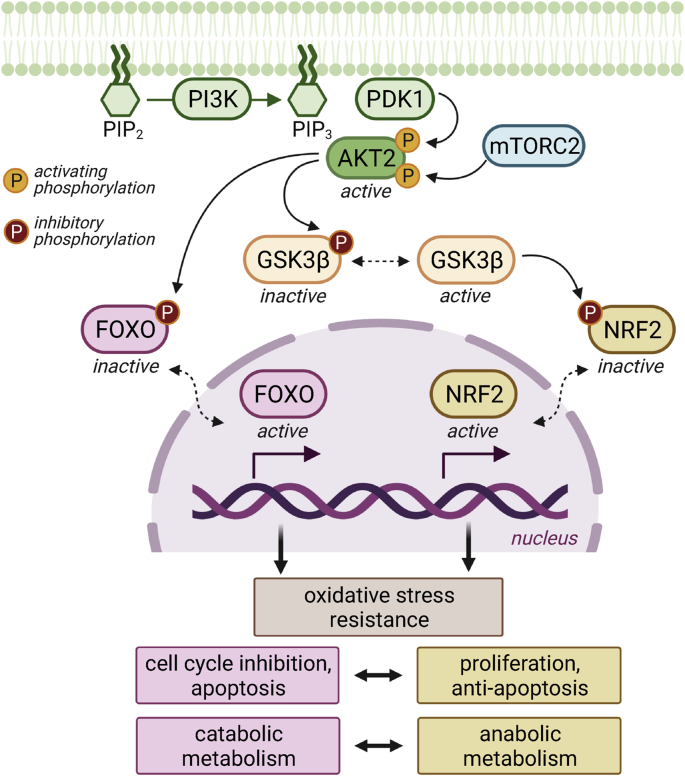
Insulin signalling regulates the activity of FOXO and NRF2. The role of insulin signalling is primarily the maintenance of glucose homeostasis and anabolic processes. GSK3β is an enzyme that counteracts insulin-stimulated glycogen formation. In addition, GSK3β inhibits NRF2 via phosphorylation and nuclear exclusion, thus impacting the generation of reducing equivalents (e.g. NADPH) necessary for anabolic reactions. However, phosphorylated (i.e. active) AKT2 leads to inhibitory phosphorylation of GSK3β, therefore leaving NRF2 active. In contrast, AKT2 also phosphorylates and inhibits FOXO, a major transcription factor regulating cellular responses to fasting.

NRF2 plays a major role in the maintenance of cellular redox homeostasis by controlling the response to oxidative stress. Under unstressed conditions, NRF2 is bound to its regulatory partner Keap1 (kelch-like ECH-associated protein 1) and targeted for proteasomal degradation, resulting in the constitutive low abundance of active NRF2. Keap1 contains multiple conserved and highly redox-sensitive cysteine residues, situated close to polar and basic amino acids, which can be modified by H_2_O_2_ or other electrophiles resulting in the altered conformation of Keap1. Upon oxidative stress or in the presence of other electrophilic compounds, oxidation of the cysteine residues in Keap1 impairs its binding to NRF2, limiting its ability to present NRF2 for proteasomal degradation. Active (released) NRF2 translocates into the nucleus, where it induces the transcription of genes containing an antioxidant response element (ARE) in their promoter regions [126]. However, NRF2 has been implicated not only in redox homeostasis, but also in DNA repair, mitochondrial function, proteostasis, and proliferation [127]. Besides this redox regulatory mechanism, NRF2 is also strongly regulated via other PTMs (e.g. phosphorylation) that prevent or promote its nuclear accumulation (Fig. 4).

Glycogen synthase kinase 3β (GSK3β) is an important regulator of glycogen metabolism by inhibiting via phosphorylation the activity of glycogen synthase. In addition, GSK3β regulates the cell cycle, as well as apoptosis and insulin signalling [128]. GSK3β is active in unstressed non-stimulated cells, and is regulated via inhibitory phosphorylation of two *N*-terminal serine residues (Ser9 and Ser21) in response to growth factors. Therefore, active AKT inhibits the activity of GSK3β.

GSK3β phosphorylates many proteins that are targets of the SCFβ-TrCP ubiquitin ligase complex, which promotes proteasomal degradation [129]. NRF2 contains two β-TrCP recognitions sites, one of which contains a functional GSK3β phosphorylation site [130]. Constitutively active GSK3β-ΔSer9 increases the ubiquitination of NRF2 and further reduces its protein levels [130]. Enhancing PTEN activity via selective drugs and genetic manipulation results in GSK3β-mediated phosphorylation of NRF2 at Ser335 and Ser338 residues, and β-TrCP-mediated NRF2 degradation [131]. A second mechanism by which GSK3β inhibits NRF2 is via the activation of tyrosine kinases. GSK3β phosphorylates and activates Fyn, which translocates to the nucleus where it phosphorylates NRF2, resulting in NRF2 nuclear export and degradation [132,133]. Further evidence for the cross-talk between PI3K/AKT and NRF2 was obtained by microarray data derived from endometrioid carcinomas, showing that PTEN-deficient tumours express high levels of NRF2 and its targets [131].

Due to its strong induction of antioxidant enzymes, the role of NRF2 in disorders such as insulin resistance has been extensively studied over recent years [[134], [135], [136]]. The activation of NRF2 can have either beneficial or harmful effects dependent on the physiological and pathological context [127]. In diseases characterised by chronic inflammation and associated with high levels of ROS, the activation of NRF2 is expected to have protective effects. However, prolonged activation of NRF2 might lead to reductive stress, the disruption of normal redox signalling events, and therefore to the progression of disease, as described for several types of cancer [24].

Prolonged activation of NRF2 may also result in metabolic changes as NADPH-generating enzymes are targets of NRF2. Therefore, NRF2 acts as a direct link between redox homeostasis and energy metabolism [137]. NRF2 tightly controls GSH biosynthesis by regulating the expression of two GCL subunits, which catalyses the rate-limiting step of GSH biosynthesis, namely the reaction of glutamate with cysteine [138]. Cysteine is generated from the reduction of cystine, which is imported into cells via an X_c_^−^ transporter. By activating *Slc7a11*, the gene coding for the subunit xCT of X_c_^−^, NRF2 also directly controls cystine import into cells [138]. In addition, many diverse GSH-dependent enzymes contain an ARE in their promotor sequence, and are regulated at the transcriptional level by NRF2 – for instance, GPXs that use GSH to detoxify H_2_O_2_, GSTs that transfer GSH to proteins or xenobiotics, or GR that is responsible for the regeneration of reduced GSH from its oxidised form GSSG [139].

NRF2 promotes the regeneration of NADPH through the induction of NADP^+^-dependent enzymes, including malic enzyme (ME1), isocitrate dehydrogenase (IDH1), glucose-6-phosphate 1-dehydrogenase (G6PD) and 6-phosphogluconate dehydrogenase (PGD), all part of the pentose phosphate pathway (PPP) [139,140]. NADPH is essential for recycling GSSG to GSH mediated by GR, as well as an important cofactor in anabolic reactions such as lipid synthesis. NRF2-KO mice show lower NADPH/NADP^+^ ratios, and NADPH levels decrease upon knock-down of NRF2 in tumour cells [141] Thus, decreased NRF2 activity not only results in lower antioxidant capacity, but also affects fundamental metabolic pathways such as lipogenesis, nucleotide biosynthesis, gluconeogenesis and β-oxidation.

As NRF2 exhibits important cytoprotective, antioxidant and anti-inflammatory effects, many studies have examined the role of NRF2 in the development of diseases including insulin resistance and T2D. The initial steps in the development of T2D are thought to be mediated by enhanced ROS formation due to hyperglycemia that exacerbates redox dysfunction [142]. NRF2-deficient mice are characterised by lower basal insulin levels and longer periods of hyperglycemia. Conversely, mice with pharmacologically activated NRF2 show lower blood glucose, improved insulin secretion, and higher insulin sensitivity [143,144], indicating anti-diabetic effects of NRF2. NRF2-KO mice also exhibit more profound diabetic complications such as retinopathy, nephropathy and cardiomyopathy compared to wild-type (WT) controls [133,[143], [144], [145], [146], [147], [148], [149]]. NRF2-deficiency can induce hepatic insulin resistance via activation of the NFκB signalling pathway in mice fed a HFD [150]. In contrast, several studies using transgenic mice found that NRF2-deficiency reduces insulin resistance and improves glucose homeostasis in HFD obesity models [151,152]. However, in a tissue-targeted NRF2-KO mouse model, a HFD enhanced insulin resistance in adipocytes, whereas hepatocytes showed improved insulin sensitivity [151].

The exact mechanisms whereby NRF2 impacts the development of insulin resistance are not fully understood, but lack of NRF2 would potentially lead to: **i)** enhanced levels of ROS due to diminished antioxidant capacity, and **ii)** impaired re-activation of inactivated PTPs due to the lack of Trx [84]. Consistent with this model, AKT phosphorylation at Ser473 was greater in the liver and skeletal muscle of NRF2-KO mice compared to their WT counterparts after intraperitoneal insulin injection [153].

### 5.2 Insulin signalling blocks the activity of FOXOs

**Fig. 5:**
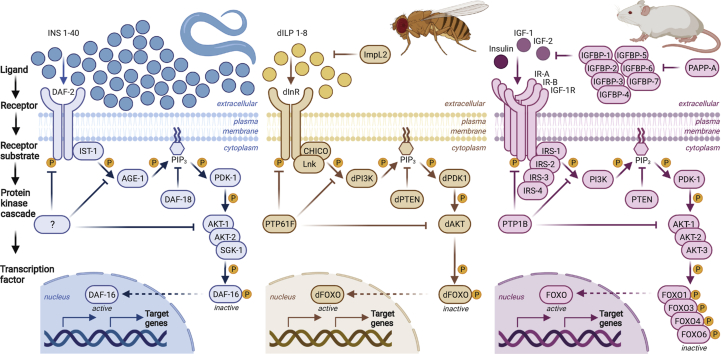
Evolutionary conservation of the insulin signalling pathway in model organisms. The binding of insulin or insulin-like ligands to the insulin receptor (IR) initiates a cascade of phosphorylation events resulting in the activation of AKT, which in turn regulates the downstream transcription factor FOXO. The termination of this signal transduction is mediated by phosphatases, which dephosphorylate and thereby inhibit the respective key players, such as AKT. The insulin signalling pathway is highly conserved between invertebrate and mammalian model organisms: the nematode worm *C. elegans*, the fruit fly *Drosophila melanogaster*, and the mouse. Some notable differences include the number of insulin and insulin-like ligands, with only 3 identified in mammals (insulin, IGF-1, and IGF-2), while worms and flies contain ~40 and 8, respectively. In contrast, downstream components of the signalling cascade (including the IR itself, AKT and FOXO) are less redundant in invertebrates. This provides an advantage to study the functionality of these components under different conditions without potential compensatory effects from paralogues.

The forkhead box class O (FOXO) family of transcription factors is named after the *Drosophila melanogaster* gene *fkh* (fork head), because mutations in this gene cause developmental defects in adult flies phenotypically appearing as a spiked head [154]. Mammals have four FOXO isoforms: FOXO1, FOXO3 and FOXO4, which share high sequence similarity, and FOXO6, which is less similar to the others members, has more restricted expression and distinct regulatory mechanisms. *C. elegans* and *Drosophila* each have only a single FOXO orthologue, called DAF-16 and dFOXO respectively (Fig. 5).

FOXOs have highly conserved functional domains: the forkhead DNA-binding domain, and domains that control nuclear import/export and transactivation. The best understood mechanism by which FOXO proteins regulate gene expression is via the evolutionarily conserved forkhead domain, a winged-helix DNA-binding domain, that recognises the core DNA motif 5′-TTGTTTAC-3′ [155]. To ensure the correct cell type-specific effect is initiated by these widely expressed transcription factors, FOXO proteins interact with a range of binding partners such as STAT3, Smads, P300, C/EBPα/β and PPARs, allowing for a much broader transcriptional response [156]. The plasticity of FOXO activity is regulated by several PTMs that determine subcellular localisation, including phosphorylation, acetylation, methylation, glycosylation, protein-protein interactions, nuclear shuttling, and redox regulation.

FOXO1 is highly expressed in metabolically active cells, such as hepatocytes and adipocytes, where it regulates the transcription of genes involved in adipocyte differentiation and trans-differentiation, oxidative stress response, lipid metabolism, and the induction of gluconeogenic genes during conditions of low energy intake [157]. Under starvation, FOXO1 responds to increasing ROS levels in adipocytes, determining the transcription of mitochondrial anti-stress response genes [158]. FOXO acts as a nutrient sensor in response to insulin signalling via nucleo-cytoplasmic shuttling, and is therefore a crucial regulator of metabolism. In this context, insulin has an inhibitory function on FOXO proteins, as active AKT phosphorylates FOXO at three sites leading to its inactivation via decreased DNA-binding to its consensus response elements, enhanced association with 14-3-3 proteins, and nuclear exclusion [157] (Fig. 4).

FOXO transcription factors regulate a wide range of genes that can be clustered in the following categories: cell fate decisions (cell cycle arrest, apoptosis), metabolism (gluconeogenesis, food intake, redox balance), protein homeostasis (mitophagy, proteasomal degradation, autophagy), signalling (increased PI3K signalling, increased EGF signalling, decreased mTORC1 signalling, increased mTORC2 signalling, decreased MYC signalling), and cell type-specific functions (pluripotent maintenance, immune system) [157]. FOXO targets include intra- and extracellular antioxidants that interfere with all levels of oxygen reduction that would otherwise lead to the formation of ROS and cause oxidative damage [159].

Hyper-activation of FOXOs is associated with hyperglycemia, hypertriglyceridemia, and insulin resistance [160,161]. Transgenic mice expressing constitutively active FOXO1 in either hepatic cells or pancreatic β-cells show increased hepatic gluconeogenesis and decreased β-cell function, respectively [162]. The activity of FOXO is upregulated upon nutrient restriction in adipocytes leading to improved antioxidant responses and lipid catabolism, as well as white-to-brown adipocyte remodelling [163].

**Fig. 6:**
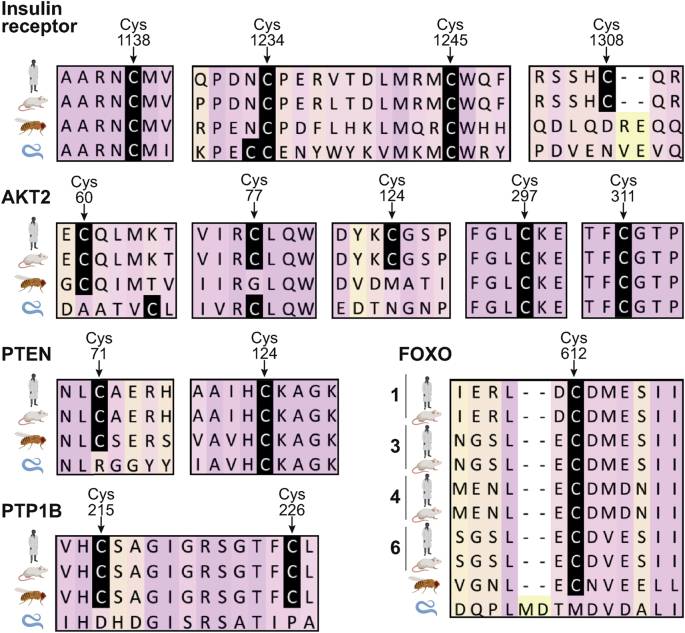
The evolutionary conversation of redox-sensitive insulin signalling components. Protein sequence alignment of insulin signalling components reveals that a high proportion of cysteines described to be redox-sensitive are conserved between mammals and invertebrates. The regions surrounding the redox-sensitive cysteines are shown, and the respective cysteines are highlighted in black (numbering corresponds to the position in the upper human sequence). The sequences were obtained from UniProt (www.uniprot.org), aligned by T-Coffee (www.ebi.ac.uk/Tools/msa/tcoffee/) [164] and analysed with JalView [165]. The UniProt IDs for human (*Hs*), mouse (*Mm*), *Drosophila melanogaster* (*Dm*) and *C. elegans* (*Ce*) are as follows: **IR:** P06213-2 (*Hs*), P15208 (*Mm*), P09208 (*Dm*), Q968Y9 (*Ce*); **PTEN:** P60484 (*Hs*), O08586 (*Mm*), Q9V0B6 (*Dm*), G5EE01 (*Ce*); **PTP1B:** P18031 (*Hs*), P35821 (*Mm*), Q8IRH4 (*Dm*), C3JXD8 (*Ce*); **AKT2:** P31751 (*Hs*), Q60823, Q8INB9 (*Dm*), Q9XTG7 (*Ce*); **FOXO1,3,4,6** (*Hs*)**:** Q12778, O43524, P98177, A8MYZ6; **FOXO1,3,4,6** (*Mm*)**:** Q9R1E0, Q9WVH4, Q9WVH3, Q70KY4; **FOXO:** Q95V55 (*Dm*), O16850 (*Ce*).

FOXO transcription factors contain several cysteine residues that are potentially prone to redox regulation in response to changes in cellular redox status. Depending on the respective redox modification, the subcellular localisation, DNA binding capacity, and/or transcriptional activity of FOXO might be altered. Recent studies have confirmed that FOXO proteins undergo redox regulation (reviewed in Ref. [159]). Initial evidence was provided by a study of FOXO4 in HEK293T cells, where all the cysteine residues in FOXO4 were mutated to redox-inert serines. Redox-inactive FOXO4-ΔCys did not bind to the acetylase p300/CBP, unlike the WT control [79]. Next, using knock-in mutants where all cysteines but one were systematically replaced by serines, Cys477 was identified as being responsible for the interaction with p300/CBP upon exogenous H_2_O_2_ treatment [79]. This cysteine is conserved amongst the different FOXO isoforms in humans and mice, as well as in invertebrates such as *Drosophila* (Fig. 6). Interestingly, Cys612 in FOXO1 (corresponding to Cys477 in FOXO4) was subsequently shown to be necessary and sufficient for full FOXO1 transactivation under unstressed conditions, and required for co-activation by CBP and PGC1α [77,76]. ROS also directly regulate the nuclear import of FOXO4 by mediating heterodimerisation with TNPO1 through an intermolecular disulphide between TNPO1 with Cys239 of FOXO4 [80]. The formation of this disulphide enhances the accumulation of FOXO4 in the nucleus, and intriguingly, this mechanism is conserved in *C. elegans* [80]. Prx1 was found to undergo disulphide-dependent heterodimerisation with FOXO3a, dependent on Cys31 and Cys150 in FOXO3a [78]. Absence of Cys31 and Cys150 diminishes FOXO3a translocation into the nucleus [78]. To-date, several cysteine residues in FOXO isoforms have been identified to be redox-sensitive (Table 1), however most of these studies were conducted in human cell culture systems combined with exogenous H_2_O_2_ treatment, and whether insulin-induced H_2_O_2_ affects redox regulation of FOXO is currently unknown.

## 6 The insulin signalling pathway including its redox-sensitive cysteines is conserved

The insulin signalling pathway is associated with metabolism and growth, and is highly evolutionarily conserved from invertebrates to mammals (Fig. 5). Indeed, many seminal discoveries in the field of insulin signalling, especially how down-regulation of this pathway is linked to longevity, were initially made in *C. elegans* and *Drosophila*, and subsequently translated to mammals [[166], [167], [168]].

The insulin signalling cascade is comprised of several phosphorylation steps that lead to the activation of AKT, which mediates the ultimate downstream events. The initial step is the binding of insulin to its receptor. Whereas worms and flies contain ~40 and 8 different insulin-like ligands, respectively, mammals have only 3: insulin, IGF-1 and IGF-2. In contrast, worms and flies each have only a single orthologue of the IR, while mammals have multiple types of IR (IR-A, IR-B, IGF-1R). Similarly, worms and flies have a more restricted number of IRS and AKT isoforms compared to mammals [169] (Fig. 5).

So far, most research dissecting the role of cysteine-mediated redox regulation within specific proteins involved in metabolism has been conducted in cell culture systems. Analysis of thousands of random PDB and ModBase structures with less than 70% sequence identity between any two proteins showed that cysteines are the least exposed amino acid residue [170]. In addition, it has been estimated that ~80% of all cysteines possess some functional importance due to their extreme pattern of conservation [21], being are either highly conserved or highly degenerated [170]. This observation was interpreted as a result of selective pressure to preserve cysteines in functionally relevant positions, and remove cysteines from other positions where they could be detrimental due to their high reactivity. Exposed and isolated cysteine residues might be direct targets for a wide range of oxidants, as they were found to be more reactive than buried and isolated ones (pK_a_ 7.4 vs pK_a_ 9.5). In conclusion, the high reactivity of cysteines appears to have shaped their extreme conservation pattern compared to other amino acids, and cysteine usage at protein surfaces is limited and avoided unless functionally important [170].

To determine whether insulin signalling orthologues of redox-sensitive targets described in the mammalian system are conserved in invertebrates, we performed protein sequence alignments (Fig. 6). Several cysteine residues shown to be redox-sensitive and important for regulating the activity of key insulin signalling components in mammals are conserved in *Drosophila* and *C. elegans*, indicating that similar redox-regulatory mechanisms are likely present.

Given the strong evolutionary conservation of both the insulin signalling pathway and redox-sensitive cysteines within pathway components, model organisms such as *C. elegans* and *Drosophila* are excellent systems to study the role of physiological ROS levels and their impact on signalling cascades *in vivo*. Indeed, flies share many metabolic features with mammals, and develop similar T2D-like phenotypes including hyperglycemia, insulin resistance and obesity when reared on a high sugar diet [171]. In *C. elegans,* down-regulation of insulin signalling lowers intracellular glucose levels, induces oxidative non-glucose metabolism, which is associated with ROS generation and lifespan extension [172]. In addition, redox proteomic analysis of adult flies revealed that nutritional status dramatically impacts the redox state of cysteines, with fasting inducing a pronounced oxidising shift compared to fed controls [173]. Further studies will be useful to dissect how fasting impacts cellular redox status, and the role played by low molecular weight thiol compounds, such as GSH, in this process.

Compared to mammals, *C. elegans* and *Drosophila* have generally fewer isoforms of the respective key players in their insulin signalling pathways (Fig. 5). This lower genetic redundancy facilitates the generation of KO, redox-null, and specific cysteine mutants without potential compensatory effects from other isoforms. In addition to the high conservation of genes identified to cause diseases in humans [174,175], counterparts of organs and tissues fulfilling metabolic functions in humans are also found in *Drosophila* [176]. Many powerful genetic tools are also available, allowing tissue-specific and spatio-temporal experiments, as well as various strategies to manipulate the antioxidant capacity and redox state of the fly (reviewed in Ref. [177]). Finally, technological advances enabling the identification and manipulation of redox-sensitive cysteines can equally be applied to the *C. elegans* and *Drosophila* model systems*.*

## 7 Conclusions and future directions

The redox modification of cysteine residues is an efficient modulator of protein function under both physiological and pathophysiological conditions. Evidence obtained from *in vitro* experiments highlights that H_2_O_2_ is essential for normal cellular phosphorylation signalling events by influencing key regulatory nodes such as AKT. Studies in humans support the physiological importance of redox signalling *in vivo* – for instance, antioxidant treatment prevents the ROS-mediated insulin-sensitising benefits of physical exercise [178].

As described, ROS affect insulin action in two ways: **i)** insulin-induced H_2_O_2_ formation is essential for mediating the insulin message to downstream targets, and **ii)** ROS are also involved in the development of insulin resistance and T2D. Therefore, combatting insulin resistance via the general manipulation of the redox system is challenging. Several interventions manipulating redox status to improve insulin action have not been as effective as anticipated (reviewed in Ref. [179]). Further work will help define the regulatory components, mechanisms and redox networks, by identifying specific cellular targets susceptible to oxidative modification by insulin-stimulated H_2_O_2_ *in vivo*. Overall, improving our understanding of how and when physiological H_2_O_2_ is involved in the regulation of insulin signalling under healthy conditions, and how H_2_O_2_ contributes to the progression of metabolic diseases will improve prospects for successful therapeutic interventions*.*

## Declaration of competing interest

The authors declare that there are no competing interests associated with the manuscript.

## References

[1] Narasimhan S.D., Yen K., Tissenbaum H.A. (2009). Converging pathways in lifespan regulation. Curr. Biol..

[2] Siddle K. (2011). Signalling by insulin and IGF receptors: supporting acts and new players. J. Mol. Endocrinol..

[3] Kolb H., Kempf K., Röhling M., Martin S. (2020). Insulin: too much of a good thing is bad. BMC Med..

[4] American Diabetes Association (2014). Diagnosis and classification of diabetes mellitus. Diabetes Care.

[5] Saeedi P., Petersohn I., Salpea P., Malanda B., Karuranga S., Unwin N., Colagiuri S., Guariguata L., Motala A.A., Ogurtsova K., Shaw J.E., Bright D., Williams R. (2019). Global and regional diabetes prevalence estimates for 2019 and projections for 2030 and 2045: results from the International Diabetes Federation Diabetes Atlas, 9^th^ edition. Diabetes Res. Clin. Pract..

[6] NCD Risk Factor Collaboration (NCD-RisC) (2016). Worldwide trends in diabetes since 1980: a pooled analysis of 751 population-based studies with 4.4 million participants. Lancet.

[7] Trikkalinou A., Papazafiropoulou A.K., Melidonis A. (2017). Diabetes and quality of life. World J. Diabetes.

[8] Roberts S.B., Das S.K., Suen V.M.M., Pihlajamäki J., Kuriyan R., Steiner-Asiedu M., Taetzsch A., Anderson A.K., Silver R.E., Barger K., Krauss A., Karhunen L., Zhang X., Hambly C., Schwab U., Triffoni-Melo A.D.T., Fassini P.G., Taylor S.F., Economos C., Kurpad A.V., Speakman J.R. (2018). Measured energy content of frequently purchased restaurant meals: multi-country cross sectional study. BMJ.

[9] Hall K.D. (2018). Did the food environment cause the obesity epidemic?. Obesity.

[10] Chaudhuri R., Krycer J.R., Fazakerley D.J., Fisher-Wellman K.H., Su Z., Hoehn K.L., Yang J.Y.H., Kuncic Z., Vafaee F., James D.E. (2018). The transcriptional response to oxidative stress is part of, but not sufficient for, insulin resistance in adipocytes. Sci. Rep..

[11] Brownlee M. (2001). Biochemistry and molecular cell biology of diabetic complications. Nature.

[12] Evans J.L., Goldfine I.D., Maddux B.A., Grodsky G.M. (2002). Oxidative stress and stress-activated signaling pathways: a unifying hypothesis of type 2 diabetes. Endocr. Rev..

[13] Chang Y.C., Chuang L.M. (2010). The role of oxidative stress in the pathogenesis of type 2 diabetes: from molecular mechanism to clinical implication. Am. J. Transl. Res..

[14] Kim J.A., Wei Y., Sowers J.R. (2008). Role of mitochondrial dysfunction in insulin resistance. Circ. Res..

[15] Parvez S., Long M.J.C., Poganik J.R., Aye Y. (2018). Redox signaling by reactive electrophiles and oxidants. Chem. Rev..

[16] Murphy M.P. (2009). How mitochondria produce reactive oxygen species. Biochem. J..

[17] Lambeth J.D. (2004). NOX enzymes and the biology of reactive oxygen. Nat. Rev. Immunol..

[18] van Dam L., Dansen T.B. (2020). Cross-talk between redox signalling and protein aggregation. Biochem. Soc. Trans..

[19] Collins Y., Chouchani E.T., James A.M., Menger K.E., Cochemé H.M., Murphy M.P. (2012). Mitochondrial redox signalling at a glance. J. Cell Sci..

[20] Cremers C.M., Jakob U. (2013). Oxidant sensing by reversible disulfide bond formation. J. Biol. Chem..

[21] Go Y.M., Chandler J.D., Jones D.P. (2015). The cysteine proteome. Free Radic. Biol. Med..

[22] Putker M., Vos H.R., van Dorenmalen K., de Ruiter H., Duran A.G., Snel B., Burgering B.M.T., Vermeulen M., Dansen T.B. (2015). Evolutionary acquisition of cysteines determines FOXO paralog-specific redox signaling. Antioxid. Redox Signal..

[23] Paulsen C.E., Carroll K.S. (2013). Cysteine-mediated redox signaling: chemistry, biology, and tools for discovery. Chem. Rev..

[24] Lennicke C., Rahn J., Lichtenfels R., Wessjohann L.A., Seliger B. (2015). Hydrogen peroxide - production, fate and role in redox signaling of tumor cells. Cell Commun. Signal..

[25] Forman H.J., Zhang H., Rinna A. (2009). Glutathione: overview of its protective roles, measurement, and biosynthesis. Mol. Aspects Med..

[26] Bindoli A., Fukuto J.M., Forman H.J. (2008). Thiol chemistry in peroxidase catalysis and redox signaling. Antioxid. Redox Signal..

[27] Mailloux R.J. (2020). Protein S-glutathionylation reactions as a global inhibitor of cell metabolism for the desensitization of hydrogen peroxide signals. Redox Biol..

[28] Poole L.B. (2015). The basics of thiols and cysteines in redox biology and chemistry. Free Radic. Biol. Med..

[29] Xiao H., Jedrychowski M.P., Schweppe D.K., Huttlin E.L., Yu Q., Heppner D.E., Li J., Long J., Mills E.L., Szpyt J., He Z., Du G., Garrity R., Reddy A., Vaites L.P., Paulo J.A., Zhang T., Gray N.S., Gygi S.P., Chouchani E.T. (2020). A quantitative tissue-specific landscape of protein redox regulation during aging. Cell.

[30] Su Z., Burchfield J.G., Yang P., Humphrey S.J., Yang G., Francis D., Yasmin S., Shin S.Y., Norris D.M., Kearney A.L., Astore M.A., Scavuzzo J., Fisher-Wellman K.H., Wang Q.P., Parker B.L., Neely G.G., Vafaee F., Chiu J., Yeo R., Hogg P.J., Fazakerley D.J., Nguyen L.K., Kuyucak S., James D.E. (2019). Global redox proteome and phosphoproteome analysis reveals redox switch in Akt. Nat. Commun..

[31] Corcoran A., Cotter T.G. (2013). Redox regulation of protein kinases. FEBS J..

[32] Antunes F., Brito P.M. (2017). Quantitative biology of hydrogen peroxide signaling. Redox Biol..

[33] Wood Z.A., Poole L.B., Karplus P.A. (2003). Peroxiredoxin evolution and the regulation of hydrogen peroxide signaling. Science.

[34] Sobotta M.C., Liou W., Stöcker S., Talwar D., Oehler M., Ruppert T., Scharf A.N.D., Dick T.P. (2015). Peroxiredoxin-2 and STAT3 form a redox relay for H_2_O_2_ signaling. Nat. Chem. Biol..

[35] Peskin A.V., Pace P.E., Winterbourn C.C. (2019). Enhanced hyperoxidation of peroxiredoxin 2 and peroxiredoxin 3 in the presence of bicarbonate/CO_2_. Free Radic. Biol. Med..

[36] Dagnell M., Cheng Q., Rizvi S.H.M., Pace P.E., Boivin B., Winterbourn C.C., Arnér E.S.J. (2019). Bicarbonate is essential for protein-tyrosine phosphatase 1B (PTP1B) oxidation and cellular signaling through EGF-triggered phosphorylation cascades. J. Biol. Chem..

[37] Hoehn K.L., Hohnen-Behrens C., Cederberg A., Wu L.E., Turner N., Yuasa T., Ebina Y., James D.E. (2008). IRS1-independent defects define major nodes of insulin resistance. Cell Metab..

[38] Till J.H., Ablooglu A.J., Frankel M., Bishop S.M., Kohanski R.A., Hubbard S.R. (2001). Crystallographic and solution studies of an activation loop mutant of the insulin receptor tyrosine kinase: insights into kinase mechanism. J. Biol. Chem..

[39] Lemmon M.A., Schlessinger J. (2010). Cell signaling by receptor tyrosine kinases. Cell.

[40] Fayard E., Xue G., Parcellier A., Bozulic L., Hemmings B.A. (2010). Protein kinase B (PKB/Akt), a key mediator of the PI3K signaling pathway. Curr. Top. Microbiol. Immunol..

[41] Dummler B., Hemmings B.A. (2007). Physiological roles of PKB/Akt isoforms in development and disease. Biochem. Soc. Trans..

[42] Cho H., Mu J., Kim J.K., Thorvaldsen J.L., Chu Q., Crenshaw E.B., Kaestner K.H., Bartolomei M.S., Shulman G.I., Birnbaum M.J. (2001). Insulin resistance and a diabetes mellitus-like syndrome in mice lacking the protein kinase Akt2 (PKBβ). Science.

[43] Zhou Q.L., Park J.G., Jiang Z.Y., Holik J.J., Mitra P., Semiz S., Guilherme A., Powelka A.M., Tang X., Virbasius J., Czech M.P. (2004). Analysis of insulin signalling by RNAi-based gene silencing. Biochem. Soc. Trans..

[44] George S., Rochford J.J., Wolfrum C., Gray S.L., Schinner S., Wilson J.C., Soos M.A., Murgatroyd P.R., Williams R.M., Acerini C.L., Dunger D.B., Barford D., Umpleby A.M., Wareham N.J., Davies H.A., Schafer A.J., Stoffel M., O'Rahilly S., Barroso I. (2004). A family with severe insulin resistance and diabetes due to a mutation in AKT2. Science.

[45] Hussain K., Challis B., Rocha N., Payne F., Minic M., Thompson A., Daly A., Scott C., Harris J., Smillie B.J.L., Savage D.B., Ramaswami U., De Lonlay P., O'Rahilly S., Barroso I., Semple R.K. (2011). An activating mutation of AKT2 and human hypoglycemia. Science.

[46] Hale A.J., Ter Steege E., den Hertog J. (2017). Recent advances in understanding the role of protein-tyrosine phosphatases in development and disease. Dev. Biol..

[47] Klaman L.D., Boss O., Peroni O.D., Kim J.K., Martino J.L., Zabolotny J.M., Moghal N., Lubkin M., Kim Y.-B., Sharpe A.H., Stricker-Krongrad A., Shulman G.I., Neel B.G., Kahn B.B. (2000). Increased energy expenditure, decreased adiposity, and tissue-specific insulin sensitivity in protein-tyrosine phosphatase 1B-deficient mice. Mol. Cell. Biol..

[48] González-Rodríguez Á., Más-Gutierrez J.A., Mirasierra M., Fernandez-Pérez A., Lee Y.J., Ko H.J., Kim J.K., Romanos E., Carrascosa J.M., Ros M., Vallejo M., Rondinone C.M., Valverde Á.M. (2012). Essential role of protein tyrosine phosphatase 1B in obesity-induced inflammation and peripheral insulin resistance during aging. Aging Cell.

[49] Kipfer-Coudreau S., Eberlé D., Sahbatou M., Bonhomme A., Guy-Grand B., Froguel P., Galan P., Basdevant A., Clément K. (2004). Single nucleotide polymorphisms of protein tyrosine phosphatase 1B gene are associated with obesity in morbidly obese French subjects. Diabetologia.

[50] Paglialunga S., Ludzki A., Root-McCaig J., Holloway G.P. (2015). In adipose tissue, increased mitochondrial emission of reactive oxygen species is important for short-term high-fat diet-induced insulin resistance in mice. Diabetologia.

[51] Boden G., Homko C., Barrero C.A., Stein T.P., Chen X., Cheung P., Fecchio C., Koller S., Merali S. (2015). Excessive caloric intake acutely causes oxidative stress, GLUT4 carbonylation, and insulin resistance in healthy men. Sci. Transl. Med..

[52] McClung J.P., Roneker C.A., Mu W., Lisk D.J., Langlais P., Liu F., Lei X.G. (2004). Development of insulin resistance and obesity in mice overexpressing cellular glutathione peroxidase. Proc. Natl. Acad. Sci. U.S.A..

[53] Goldstein B.J., Mahadev K., Wu X., Zhu L., Motoshima H. (2005). Role of insulin-induced reactive oxygen species in the insulin signaling pathway. Antioxid. Redox Signal..

[54] Contreras-Ferrat A., Llanos P., Vásquez C., Espinosa A., Osorio-Fuentealba C., Arias-Calderon M., Lavandero S., Klip A., Hidalgo C., Jaimovich E. (2014). Insulin elicits a ROS-activated and an IP3-dependent Ca^2+^ release, which both impinge on GLUT4 translocation. J. Cell Sci..

[55] Loh K., Deng H., Fukushima A., Cai X., Boivin B., Galic S., Bruce C., Shields B.J., Skiba B., Ooms L.M., Stepto N., Wu B., Mitchell C.A., Tonks N.K., Watt M.J., Febbraio M.A., Crack P.J., Andrikopoulos S., Tiganis T. (2009). Reactive oxygen species enhance insulin sensitivity. Cell Metab..

[56] May J.M., Haën C. (1979). Insulin-stimulated intracellular hydrogen peroxide production in rat epididymal fat cells. J. Biol. Chem..

[57] Mahadev K., Wu X., Zilbering A., Zhu L., Lawrence J.T.R., Goldstein B.J. (2001). Hydrogen peroxide generated during cellular insulin stimulation is integral to activation of the distal insulin signaling cascade in 3T3-L1 adipocytes. J. Biol. Chem..

[58] Bedard K., Krause K.H. (2007). The NOX family of ROS-generating NADPH oxidases: physiology and pathophysiology. Physiol. Rev..

[59] Mahadev K., Motoshima H., Wu X., Ruddy J.M., Arnold R.S., Cheng G., Lambeth J.D., Goldstein B.J. (2004). The NAD(P)H oxidase homolog Nox4 modulates insulin-stimulated generation of H_2_O_2_ and plays an integral role in insulin signal transduction. Mol. Cell. Biol..

[60] Steinhorn B., Sartoretto J.L., Sorrentino A., Romero N., Kalwa H., Abel E.D., Michel T. (2017). Insulin-dependent metabolic and inotropic responses in the heart are modulated by hydrogen peroxide from NADPH-oxidase isoforms NOX2 and NOX4. Free Radic. Biol. Med..

[61] Li Y., Mouche S., Sajic T., Veyrat-Durebex C., Supale R., Pierroz D., Ferrari S., Negro F., Hasler U., Feraille E., Moll S., Meda P., Deffert C., Montet X., Krause K.H., Szanto I. (2012). Deficiency in the NADPH oxidase 4 predisposes towards diet-induced obesity. Int. J. Obes..

[62] Park H.S., Jin D.K., Shin S.M., Jang M.K., Longo N., Park J.W., Bae D.S., Bae Y.S. (2005). Impaired generation of reactive oxygen species in leprechaunism through downregulation of Nox4. Diabetes.

[63] Furukawa S., Fujita T., Shimabukuro M., Iwaki M., Yamada Y., Nakajima Y., Nakayama O., Makishima M., Matsuda M., Shimomura I. (2004). Increased oxidative stress in obesity and its impact on metabolic syndrome. J. Clin. Invest..

[64] Anvari E., Wikström P., Walum E., Welsh N. (2015). The novel NADPH oxidase 4 inhibitor GLX351322 counteracts glucose intolerance in high-fat diet-treated C57BL/6 mice. Free Radic. Res..

[65] Clark S., Konstantopoulos N. (1993). Sulphydryl agents modulate insulin- and epidermal growth factor (EGF)-receptor kinase via reaction with intracellular receptor domains: differential effects on basal versus activated receptors. Biochem. J..

[66] Schmid E., El Benna J., Galter D., Klein G., Dröge W. (1998). Redox priming of the insulin receptor β‐chain associated with altered tyrosine kinase activity and insulin responsiveness in the absence of tyrosine autophosphorylation. FASEB J..

[67] Schmid E., Hotz‐Wagenblatt A., Hack V., Dröge W. (1999). Phosphorylation of the insulin receptor kinase by phosphocreatine in combination with hydrogen peroxide: the structural basis of redox priming. FASEB J..

[68] Schmitt T.L., Hotz-Wagenblatt A., Klein H., Dröge W. (2005). Interdependent regulation of insulin receptor kinase activity by ADP and hydrogen peroxide. J. Biol. Chem..

[69] Lee S.R., Yang K.S., Kwon J., Lee C., Jeong W., Rhee S.G. (2002). Reversible inactivation of the tumor suppressor PTEN by H_2_O_2_. J. Biol. Chem..

[70] Kwon J., Lee S.R., Yang K.S., Ahn Y., Kim Y.J., Stadtman E.R., Rhee S.G. (2004). Reversible oxidation and inactivation of the tumor suppressor PTEN in cells stimulated with peptide growth factors. Proc. Natl. Acad. Sci. U.S.A..

[71] Seo J.H., Ahn Y., Lee S.-R., Yeo C.Y., Hur K.C. (2005). The major target of the endogenously generated reactive oxygen species in response to insulin stimulation is phosphatase and tensin homolog and not phosphoinositide-3 kinase (PI-3 kinase) in the PI-3 kinase/Akt pathway. Mol. Biol. Cell.

[72] Salmeen A., Andersen J.N., Myers M.P., Meng T.C., Hinks J.A., Tonks N.K., Barford D. (2003). Redox regulation of protein tyrosine phosphatase 1B involves a sulphenyl-amide intermediate. Nature.

[73] Wani R., Qian J., Yin L., Bechtold E., King S.B., Poole L.B., Paek E., Tsang A.W., Furdui C.M. (2011). Isoform-specific regulation of Akt by PDGF-induced reactive oxygen species. Proc. Natl. Acad. Sci. U.S.A..

[74] Huang X., Begley M., Morgenstern K.A., Gu Y., Rose P., Zhao H., Zhu X. (2003). Crystal structure of an inactive Akt2 kinase domain. Structure.

[75] Murata H., Ihara Y., Nakamura H., Yodoi J., Sumikawa K., Kondo T. (2003). Glutaredoxin exerts an antiapoptotic effect by regulating the redox state of Akt. J. Biol. Chem..

[76] Tsitsipatis D., Gopal K., Steinbrenner H., Klotz L.O. (2018). FOXO1 cysteine-612 mediates stimulatory effects of the coregulators CBP and PGC1α on FOXO1 basal transcriptional activity. Free Radic. Biol. Med..

[77] Sewastianik T., Szydlowski M., Jablonska E., Bialopiotrowicz E., Kiliszek P., Gorniak P., Polak A., Prochorec-Sobieszek M., Szumera-Cieckiewicz A., Kaminski T.S., Markowicz S., Nowak E., Grygorowicz M.A., Warzocha K., Juszczynski P. (2016). FOXO1 is a TXN- and p300-dependent sensor and effector of oxidative stress in diffuse large B-cell lymphomas characterized by increased oxidative metabolism. Oncogene.

[78] Hopkins B.L., Nadler M., Skoko J.J., Bertomeu T., Pelosi A., Shafaei P.M., Levine K., Schempf A., Pennarun B., Yang B., Datta D., Bucur O., Ndebele K., Oesterreich S., Yang D., Giulia Rizzo M., Khosravi-Far R., Neumann C.A. (2018). A peroxidase peroxiredoxin 1-specific redox regulation of the novel FOXO3 microRNA target let-7. Antioxid. Redox Signal..

[79] Dansen T.B., Smits L.M.M., Van Triest M.H., De Keizer P.L.J., Van Leenen D., Koerkamp M.G., Szypowska A., Meppelink A., Brenkman A.B., Yodoi J., Holstege F.C.P., Burgering B.M.T. (2009). Redox-sensitive cysteines bridge p300/CBP-mediated acetylation and FoxO4 activity. Nat. Chem. Biol..

[80] Putker M., Madl T., Vos H.R., de Ruiter H., Visscher M., van den Berg M.C.W., Kaplan M., Korswagen H.C., Boelens R., Vermeulen M., Burgering B.M.T., Dansen T.B. (2013). Redox-dependent control of FOXO/DAF-16 by transportin-1. Mol. Cell.

[81] Kowalski A., Gazzano H., Fehlmann M., Van Obberghen E. (1983). Dephosphorylation of the hepatic insulin receptor: absence of intrinsic phosphatase activity in purified receptors. Biochem. Biophys. Res. Commun..

[82] Haring H.U., Kasuga M., White M.F., Crettaz M., Kahn C.R. (1984). Phosphorylation and dephosphorylation of the insulin receptor: evidence against an intrinsic phosphatase activity. Biochemistry.

[83] Barrett W.C., DeGnore J.P., Keng Y.F., Zhang Z.Y., Yim M.B., Chock P.B. (1999). Roles of superoxide radical anion in signal transduction mediated by reversible regulation of protein-tyrosine phosphatase 1B. J. Biol. Chem..

[84] Dagnell M., Frijhoff J., Pader I., Augsten M., Boivin B., Xu J., Mandal P.K., Tonks N.K., Hellberg C., Conrad M., Arner E.S.J., Ostman A. (2013). Selective activation of oxidized PTP1B by the thioredoxin system modulates PDGF-β receptor tyrosine kinase signaling. Proc. Natl. Acad. Sci. U.S.A..

[85] Lee J.O., Yang H., Georgescu M.M., Di Cristofano A., Maehama T., Shi Y., Dixon J.E., Pandolfi P., Pavletich N.P. (1999). Crystal structure of the PTEN tumor suppressor: implications for its phosphoinositide phosphatase activity and membrane association. Cell.

[86] Li D.M., Sun H. (1997). TEP1, encoded by a candidate tumor suppressor locus, is a novel protein tyrosine phosphatase regulated by transforming growth factor β. Cancer Res..

[87] Li J., Yen C., Liaw D., Podsypanina K., Bose S., Wang S.I., Puc J., Miliaresis C., Rodgers L., McCombie R., Bigner S.H., Giovanella B.C., Ittmann M., Tycko B., Hibshoosh H., Wigler M.H., Parsons R. (1997). PTEN, a putative protein tyrosine phosphatase gene mutated in human brain, breast, and prostate cancer. Science.

[88] Maehama T., Taylor G.S., Dixon J.E. (2001). PTEN and myotubularin: novel phosphoinositide phosphatases. Annu. Rev. Biochem..

[89] Leslie N.R., Bennett D., Lindsay Y.E., Stewart H., Gray A., Downes C.P. (2003). Redox regulation of PI 3-kinase signalling via inactivation of PTEN. EMBO J..

[90] Kim Y., Song Y.B., Kim T.Y., Kim I., Han S.J., Ahn Y., Cho S.H., Choi C.Y., Chay K.O., Yang S.Y., Ahn B.W., Huh W.K., Lee S.R. (2010). Redox regulation of the tumor suppressor PTEN by glutathione. FEBS Lett..

[91] Ross S.H., Lindsay Y., Safrany S.T., Lorenzo O., Villa F., Toth R., Clague M.J., Downes C.P., Leslie N.R. (2007). Differential redox regulation within the PTP superfamily. Cell. Signal..

[92] Kim J.H., Park S.J., Chae U., Seong J., Lee H.S., Lee S.R., Lee S., Lee D.S. (2018). Peroxiredoxin 2 mediates insulin sensitivity of skeletal muscles through regulation of protein tyrosine phosphatase oxidation. Int. J. Biochem. Cell Biol..

[93] Mueller A.S., Bosse A.C., Most E., Klomann S.D., Schneider S., Pallauf J. (2009). Regulation of the insulin antagonistic protein tyrosine phosphatase 1B by dietary Se studied in growing rats. J. Nutr. Biochem..

[94] Koren S., Fantus I.G. (2007). Inhibition of the protein tyrosine phosphatase PTP1B: potential therapy for obesity, insulin resistance and type-2 diabetes mellitus. Best Pract. Res. Clin. Endocrinol. Metab..

[95] Elchebly M., Payette P., Michaliszyn E., Cromlish W., Collins S., Loy A.L., Normandin D., Cheng A., Himms-Hagen J., Chan C.C., Ramachandran C., Gresser M.J., Tremblay M.L., Kennedy B.P. (1999). Increased insulin sensitivity and obesity resistance in mice lacking the protein tyrosine phosphatase-1B gene. Science.

[96] Klaman L.D., Boss O., Peroni O.D., Kim J.K., Martino J.L., Zabolotny J.M., Moghal N., Lubkin M., Kim Y.B., Sharpe A.H., Stricker-Krongrad A., Shulman G.I., Neel B.G., Kahn B.B. (2000). Increased energy expenditure, decreased adiposity, and tissue-specific insulin sensitivity in protein-tyrosine phosphatase 1B-deficient mice. Mol. Cell. Biol..

[97] Ali M.I., Ketsawatsomkron P., Belin de Chantelemele E.J., James D., Muta K., Salet C., Black S.M., Tremblay M.L., David J., Ph D., Marrero M.B., Stepp D.W. (2009). Deletion of protein tyrosin phosphatase 1B improves peripheral insulin resistance and vascular function in obese, leptin resistant mice via reduced oxidant tone. Circ. Res..

[98] Ostman A., Frijhoff J., Sandin A., Böhmer F.D. (2011). Regulation of protein tyrosine phosphatases by reversible oxidation. J. Biochem..

[99] Popov D. (2012). Endoplasmic reticulum stress and the on site function of resident PTP1B. Biochem. Biophys. Res. Commun..

[100] Boubekeur S., Boute N., Pagesy P., Zilberfarb V., Christeff N., Issad T. (2011). A new highly efficient substrate-trapping mutant of protein tyrosine phosphatase 1B (PTP1B) reveals full autoactivation of the insulin receptor precursor. J. Biol. Chem..

[101] Issad T., Boute N., Boubekeur S., Lacasa D. (2005). Interaction of PTPB with the insulin receptor precursor during its biosynthesis in the endoplasmic reticulum. Biochimie.

[102] Bettaieb A., Bakke J., Nagata N., Matsuo K., Xi Y., Liu S., Aboubechara D., Melhem R., Stanhope K., Cummings B., Graham J., Bremer A., Zhang S., Lyssiotis C.A., Zhang Z.Y., Cantley L.C., Havel P.J., Haj F.G. (2013). Protein tyrosine phosphatase 1B regulates pyruvate kinase M2 tyrosine phosphorylation. J. Biol. Chem..

[103] Tonks N.K. (2006). Protein tyrosine phosphatases: from genes, to function, to disease. Nat. Rev. Mol. Cell Biol..

[104] Ferrer-Sueta G., Manta B., Botti H., Radi R., Trujillo M., Denicola A. (2011). Factors affecting protein thiol reactivity and specificity in peroxide reduction. Chem. Res. Toxicol..

[105] Barrett W.C., DeGnore J.P., König S., Fales H.M., Keng Y.F., Zhang Z.Y., Yim M.B., Chock P.B. (1999). Regulation of PTP1B via glutathionylation of the active site cysteine 215. Biochemistry.

[106] Townsend D.M., Findlay V.J., Fazilev F., Ogle M., Fraser J., Saavedra J.E., Ji X., Keefer L.K., Tew K.D. (2006). A glutathione S-transferase π-activated prodrug causes kinase activation concurrent with S-glutathionylation of proteins. Mol. Pharmacol..

[107] Denu J.M., Tanner K.G. (1998). Specific and reversible inactivation of protein tyrosine phosphatases by hydrogen peroxide: evidence for a sulfenic acid intermediate and implications for redox regulation. Biochemistry.

[108] Juarez J.C., Manuia M., Burnett M.E., Betancourt O., Boivin B., Shaw D.E., Tonks N.K., Mazar A.P., Doñate F. (2008). Superoxide dismutase 1 (SOD1) is essential for H_2_O_2_-mediated oxidation and inactivation of phosphatases in growth factor signaling. Proc. Natl. Acad. Sci. U.S.A..

[109] Forman H.J., Maiorino M., Ursini F. (2010). Signaling functions of reactive oxygen species. Biochemistry.

[110] Tanner J.J., Parsons Z.D., Cummings A.H., Zhou H., Gates K.S. (2011). Redox regulation of protein tyrosine phosphatases: structural and chemical aspects. Antioxid. Redox Signal..

[111] Londhe A.D., Bergeron A., Curley S.M., Zhang F., Rivera K.D., Kannan A., Coulis G., Rizvi S.H.M., Kim S.J., Pappin D.J., Tonks N.K., Linhardt R.J., Boivin B. (2020). Regulation of PTP1B activation through disruption of redox-complex formation. Nat. Chem. Biol..

[112] Shimizu S., Ugi S., Maegawa H., Egawa K., Nishio Y., Yoshizaki T., Shi K., Nagai Y., Morino K., Nemoto K., Nakamura T., Bryer-Ash M., Kashiwagi A. (2003). Protein-tyrosine phosphatase 1B as new activator for hepatic lipogenesis via sterol regulatory element-binding protein-1 gene expression. J. Biol. Chem..

[113] Ferré P., Foufelle F. (2007). SREBP-1c transcription factor and lipid homeostasis: clinical perspective. Horm. Res..

[114] Schmitz-Peiffer C. (2000). Signalling aspects of insulin resistance in skeletal muscle: mechanisms induced by lipid oversupply. Cell. Signal..

[115] Diraison F., Parton L., Ferré P., Foufelle F., Briscoe C.P., Leclerc I., Rutter G.A. (2004). Over-expression of sterol-regulatory-element-binding protein-1c (SREBP1c) in rat pancreatic islets induces lipogenesis and decreases glucose- stimulated insulin release: modulation by 5-aminoimidazole- 4-carboxamide ribonucleoside (AICAR). Biochem. J..

[116] Wang H., Maechler P., Antinozzi P.A., Herrero L., Hagenfeldt-Johansson K.A., Björklund A., Wollheim C.B. (2003). The transcription factor SREBP-1c is instrumental in the development of β-cell dysfunction. J. Biol. Chem..

[117] Krishnan N., Bonham C.A., Rus I.A., Shrestha O.K., Gauss C.M., Haque A., Tocilj A., Joshua-Tor L., Tonks N.K. (2018). Harnessing insulin- and leptin-induced oxidation of PTP1B for therapeutic development. Nat. Commun..

[118] Galbo T., Perry R.J., Nishimura E., Samuel V.T., Quistorff B., Shulman G.I. (2013). PP2A inhibition results in hepatic insulin resistance despite Akt2 activation. Aging (Albany NY).

[119] Rao R.K., Clayton L.W. (2002). Regulation of protein phosphatase 2A by hydrogen peroxide and glutathionylation. Biochem. Biophys. Res. Commun..

[120] Guy G.R., Cairns J., Ng S.B., Tan Y.H. (1993). Inactivation of a redox-sensitive protein phosphatase during the early events of tumor necrosis factor/interleukin-1 signal transduction. J. Biol. Chem..

[121] Zheng X., Cartee G.D. (2016). Insulin-induced effects on the subcellular localization of AKT1, AKT2 and AS160 in rat skeletal muscle. Sci. Rep..

[122] Altomare D.A., Lyons G.E., Mitsuuchi Y., Cheng J.Q., Testa J.R. (1998). Akt2 mRNA is highly expressed in embryonic brown fat and the AKT2 kinase is activated by insulin. Oncogene.

[123] Yang J., Cron P., Thompson V., Good V.M., Hess D., Hemmings B.A., Barford D. (2002). Molecular mechanism for the regulation of protein kinase B/Akt by hydrophobic motif phosphorylation. Mol. Cell.

[124] Ebner M., Lučić I., Leonard T.A., Yudushkin I. (2017). PI(3,4,5)P_3_ engagement restricts Akt activity to cellular membranes. Mol. Cell.

[125] Wang X., Tao L., Hai C.X. (2012). Redox-regulating role of insulin: the essence of insulin effect. Mol. Cell. Endocrinol..

[126] Brigelius-Flohé R., Flohé L. (2011). Basic principles and emerging concepts in the redox control of transcription factors. Antioxid. Redox Signal..

[127] Dodson M., de la Vega M.R., Cholanians A.B., Schmidlin C.J., Chapman E., Zhang D.D. (2019). Modulating NRF2 in disease: timing is everything. Annu. Rev. Pharmacol. Toxicol..

[128] Cohen P., Frame S. (2001). The renaissance of GSK3. Nat. Rev. Mol. Cell Biol..

[129] Wu G., He X. (2006). Threonine 41 in β-catenin serves as a key phosphorylation relay residue in β-catenin degradation. Biochemistry.

[130] Chowdhry S., Zhang Y., McMahon M., Sutherland C., Cuadrado A., Hayes J.D. (2013). Nrf2 is controlled by two distinct β-TrCP recognition motifs in its Neh6 domain, one of which can be modulated by GSK-3 activity. Oncogene.

[131] Rojo A.I., Rada P., Mendiola M., Ortega-Molina A., Wojdyla K., Rogowska-Wrzesinska A., Hardisson D., Serrano M., Cuadrado A. (2014). The PTEN/NRF2 axis promotes human carcinogenesis. Antioxid. Redox Signal..

[132] Jain A.K., Jaiswal A.K. (2007). GSK-3β acts upstream of Fyn kinase in regulation of nuclear export and degradation of NF-E2 related factor 2. J. Biol. Chem..

[133] Kaspar J.W., Jaiswal A.K. (2011). Tyrosine phosphorylation controls nuclear export of Fyn, allowing Nrf2 activation of cytoprotective gene expression. FASEB J..

[134] Yagishita Y., Uruno A., Fukutomi T., Saito R., Saigusa D., Pi J., Fukamizu A., Sugiyama F., Takahashi S., Yamamoto M. (2017). Nrf2 improves leptin and insulin resistance provoked by hypothalamic oxidative stress. Cell Rep..

[135] Matzinger M., Fischhuber K., Heiss E.H. (2018). Activation of Nrf2 signaling by natural products-can it alleviate diabetes?. Biotechnol. Adv..

[136] Egea J., González-Rodríguez Á., Gómez-Guerrero C., Moreno J.A. (2019). Editorial: role of Nrf2 in disease: novel molecular mechanisms and therapeutic approaches. Front. Pharmacol..

[137] Li S., Eguchi N., Lau H., Ichii H. (2020). The role of the Nrf2 signaling in obesity and insulin resistance. Int. J. Mol. Sci..

[138] Tonelli C., Chio I.I.C., Tuveson D.A. (2018). Transcriptional regulation by Nrf2. Antioxid. Redox Signal..

[139] Tebay L.E., Robertson H., Durant S.T., Vitale S.R., Penning T.M., Dinkova-Kostova A.T., Hayes J.D. (2015). Mechanisms of activation of the transcription factor Nrf2 by redox stressors, nutrient cues, and energy status and the pathways through which it attenuates degenerative disease. Free Radic. Biol. Med..

[140] Thimmulappa R.K., Mai K.H., Srisuma S., Kensler T.W., Yamamoto M., Biswal S. (2002). Identification of Nrf2-regulated genes induced by the chemopreventive agent sulforaphane by oligonucleotide microarray. Cancer Res..

[141] Singh A., Happel C., Manna S.K., Acquaah-Mensah G., Carrerero J., Kumar S., Nasipuri P., Krausz K.W., Wakabayashi N., Dewi R., Boros L.G., Gonzalez F.J., Gabrielson E., Wong K.K., Girnun G., Biswal S. (2013). Transcription factor NRF2 regulates miR-1 and miR-206 to drive tumorigenesis. J. Clin. Invest..

[142] Cheng X., Siow R.C.M., Mann G.E. (2011). Impaired redox signaling and antioxidant gene expression in endothelial cells in diabetes: a role for mitochondria and the nuclear factor-E2-related factor 2-Kelch-like ECH-associated protein 1 defense pathway. Antioxid. Redox Signal..

[143] Aleksunes L.M., Reisman S.A., Yeager R.L., Goedken M.J., Klaassen C.D. (2010). Nuclear factor erythroid 2-related factor 2 deletion impairs glucose tolerance and exacerbates hyperglycemia in type 1 diabetic mice. J. Pharmacol. Exp. Ther..

[144] Uruno A., Furusawa Y., Yagishita Y., Fukutomi T., Muramatsu H., Negishi T., Sugawara A., Kensler T.W., Yamamoto M. (2013). The Keap1-Nrf2 system prevents onset of diabetes mellitus. Mol. Cell. Biol..

[145] Li B., Liu S., Miao L., Cai L. (2012). Prevention of diabetic complications by activation of Nrf2: diabetic cardiomyopathy and nephropathy. Exp. Diabetes Res..

[146] Xu Z., Wei Y., Gong J., Cho H., Park J.K., Sung E.R., Huang H., Wu L., Eberhart C., Handa J.T., Du Y., Kern T.S., Thimmulappa R., Barber A.J., Biswal S., Duh E.J. (2014). NRF2 plays a protective role in diabetic retinopathy in mice. Diabetologia.

[147] Cui W., Bai Y., Miao X., Luo P., Chen Q., Tan Y., Rane M.J., Miao L., Cai L. (2012). Prevention of diabetic nephropathy by sulforaphane: possible role of Nrf2 upregulation and activation. Oxid. Med. Cell. Longev..

[148] Jiang T., Huang Z., Lin Y., Zhang Z., Fang D., Zhang D.D. (2010). The protective role of Nrf2 in streptozotocin-induced diabetic nephropathy. Diabetes.

[149] Wu J., Sun X., Jiang Z., Jiang J., Xu L., Tian A., Sun X., Meng H., Li Y., Huang W., Jia Y., Wu H. (2020). Protective role of NRF2 in macrovascular complications of diabetes. J. Cell. Mol. Med..

[150] Liu Z., Dou W., Ni Z., Wen Q., Zhang R., Qin M., Wang X., Tang H., Cao Y., Wang J., Zhao S. (2016). Deletion of Nrf2 leads to hepatic insulin resistance via the activation of NF-κB in mice fed a high-fat diet. Mol. Med. Rep..

[151] Pi J., Leung L., Xue P., Wang W., Hou Y., Liu D., Yehuda-Shnaidman E., Lee C., Lau J., Kurtz T.W., Chan J.Y. (2010). Deficiency in the nuclear factor E2-related factor-2 transcription factor results in impaired adipogenesis and protects against diet-induced obesity. J. Biol. Chem..

[152] Chartoumpekis D.V., Ziros P.G., Psyrogiannis A.I., Papavassiliou A.G., Kyriazopoulou V.E., Sykiotis G.P., Habeos I.G. (2011). Nrf2 represses FGF21 during long-term high-fat diet-induced obesity in mice. Diabetes.

[153] Meakin P.J., Chowdhry S., Sharma R.S., Ashford F.B., Walsh S.V., McCrimmon R.J., Dinkova-Kostova A.T., Dillon J.F., Hayes J.D., Ashford M.L.J. (2014). Susceptibility of Nrf2-null mice to steatohepatitis and cirrhosis upon consumption of a high-fat diet is associated with oxidative stress, perturbation of the unfolded protein response, and disturbance in the expression of metabolic enzymes but not with insulin resistance. Mol. Cell. Biol..

[154] Weigel D., Jürgens G., Küttner F., Seifert E., Jäckle H. (1989). The homeotic gene fork head encodes a nuclear protein and is expressed in the terminal regions of the *Drosophila* embryo. Cell.

[155] Furuyama T., Nakazawa T., Nakano I., Mori N. (2000). Identification of the differential distribution patterns of mRNAs and consensus binding sequences for mouse DAF-16 homologues. Biochem. J..

[156] van der Vos K.E., Coffer P.J. (2008). FOXO-binding partners: it takes two to tango. Oncogene.

[157] Eijkelenboom A., Burgering B.M.T. (2013). FOXOs: signalling integrators for homeostasis maintenance. Nat. Rev. Mol. Cell Biol..

[158] Lettieri-Barbato D., Ioannilli L., Aquilano K., Ciccarone F., Rosina M., Ciriolo M.R. (2019). FoxO1 localizes to mitochondria of adipose tissue and is affected by nutrient stress. Metabolism.

[159] Klotz L.O., Sánchez-Ramos C., Prieto-Arroyo I., Urbánek P., Steinbrenner H., Monsalve M. (2015). Redox regulation of FoxO transcription factors. Redox Biol..

[160] Barthel A., Schmoll D., Unterman T.G. (2005). FoxO proteins in insulin action and metabolism. Trends Endocrinol. Metab..

[161] Gross D.N., van den Heuvel A.P.J., Birnbaum M.J. (2008). The role of FoxO in the regulation of metabolism. Oncogene.

[162] Nakae J., Biggs W.H., Kitamura T., Cavenee W.K., Wright C.V.E., Arden K.C., Accili D. (2002). Regulation of insulin action and pancreatic β-cell function by mutated alleles of the gene encoding forkhead transcription factor Foxo1. Nat. Genet..

[163] Barbato D.L., Tatulli G., Aquilano K., Ciriolo M.R. (2015). Mitochondrial Hormesis links nutrient restriction to improved metabolism in fat cell. Aging (Albany NY).

[164] Notredame C., Higgins D.G., Heringa J. (2000). T-Coffee: a novel method for fast and accurate multiple sequence alignment. J. Mol. Biol..

[165] Clamp M., Cuff J., Searle S.M., Barton G.J. (2004). The Jalview Java alignment editor. Bioinformatics.

[166] Partridge L., Alic N., Bjedov I., Piper M.D.W. (2011). Ageing in *Drosophila*: the role of the insulin/Igf and TOR signalling network. Exp. Gerontol..

[167] Kenyon C. (2011). The first long-lived mutants: discovery of the insulin/IGF-1 pathway for ageing. Philos. Trans. R. Soc. B Biol. Sci..

[168] Kenyon C. (2005). The plasticity of aging: insights from long-lived mutants. Cell.

[169] Alic N., Partridge L. (2011). Death and dessert: nutrient signalling pathways and ageing. Curr. Opin. Cell Biol..

[170] Marino S.M., Gladyshev V.N. (2010). Cysteine function governs its conservation and degeneration and restricts its utilization on protein surfaces. J. Mol. Biol..

[171] van Dam E., van Leeuwen L.A.G., dos Santos E., James J., Best L., Lennicke C., Vincent A.J., Marinos G., Foley A., Buricova M., Mokochinski J.B., Kramer H.B., Lieb W., Laudes M., Franke A., Kaleta C., Cochemé H.M. (2020). Sugar-induced obesity and insulin resistance are uncoupled from shortened survival in *Drosophila*. Cell Metab..

[172] Zarse K., Schmeisser S., Groth M., Priebe S., Beuster G., Kuhlow D., Guthke R., Platzer M., Kahn C.R., Ristow M. (2012). Impaired insulin/IGF1 signaling extends life span by promoting mitochondrial L-proline catabolism to induce a transient ROS signal. Cell Metab..

[173] Menger K.E., James A.M., Cochemé H.M., Harbour M.E., Chouchani E.T., Ding S., Fearnley I.M., Partridge L., Murphy M.P. (2015). Fasting, but not aging, dramatically alters the redox status of cysteine residues on proteins in *Drosophila melanogaster*. Cell Rep..

[174] Musselman L.P., Kühnlein R.P. (2018). *Drosophila* as a model to study obesity and metabolic disease. J. Exp. Biol..

[175] Regan J.C., Partridge L. (2013). Gender and longevity: why do men die earlier than women? Comparative and experimental evidence. Best Pract. Res. Clin. Endocrinol. Metab..

[176] Rajan A., Perrimon N. (2013). Of flies and men: insights on organismal metabolism from fruit flies. BMC Biol..

[177] Lennicke C., Cochemé H.M. (2020). Redox signalling and ageing: insights from *Drosophila*. Biochem. Soc. Trans..

[178] Ristow M., Zarse K., Oberbach A., Klöting N., Birringer M., Kiehntopf M., Stumvoll M., Kahn C.R., Blüher M. (2009). Antioxidants prevent health-promoting effects of physical exercise in humans. Proc. Natl. Acad. Sci. U.S.A..

[179] Bashan N., Kovsan J., Kachko I., Ovadia H., Rudich A. (2009). Positive and negative regulation of insulin signaling by reactive oxygen and nitrogen species. Physiol. Rev..

